# Solid-phase microextraction-based cuticular hydrocarbon profiling for intraspecific delimitation in *Acyrthosiphon pisum*

**DOI:** 10.1371/journal.pone.0184243

**Published:** 2017-08-31

**Authors:** Nan Chen, Yu Bai, Yong-Liang Fan, Tong-Xian Liu

**Affiliations:** State Key Laboratory of Crop Stress Biology for Arid Areas, and Key Laboratory of Integrated Pest Management on the Loess Plateau of Ministry of Agriculture, Northwest A&F University, Yangling, Shaanxi, China; University of Arkansas, UNITED STATES

## Abstract

Insect cuticular hydrocarbons (CHCs) play critical roles in reducing water loss and chemical communication. Species-specific CHC profiles have been used increasingly as an excellent character for species classification. However, considerably less is known about their potential for population delimitation within species. The aims of this study were to develop a solid-phase microextraction (SPME)-based CHC collection method and to investigate whether CHC profiles could serve as potential chemotaxonomic tools for intraspecific delimitation in *Acyrthosiphon pisum*. Optimization of fibers for SPME sampling revealed that 7 μm polydimethylsiloxane (PDMS) demonstrated the most efficient adsorption of CHCs among five different tested fibers. SPME sampling showed good reproducibility with repeated collections of CHCs from a single aphid. Validation of SPME was performed by comparing CHC profiles with those from conventional hexane extractions. The two methods showed no qualitative differences in CHCs, although SPME appeared to extract relatively fewer short-chained CHCs. While CHC profiles of a given population differed among developmental stages, wing dimorphism types, and host plants, wingless adult aphids showed very low variance in relative proportions of individual CHC components. Reproducibility of CHC profiles was explored further to classify wingless adult morphs of *A*. *pisum* from five different geographic regions that showed no variation in mitochondrial COI gene sequences. Our results demonstrate that CHC profiles are useful in intraspecific delimitation in the field of insect chemotaxonomy.

## Introduction

Insect cuticular hydrocarbons (CHCs) are non-polar lipids that function primarily as a barrier against desiccation, serve as species, colony, and gender-specific chemical communication cues [1,2], and are involved in insecticide resistance [3]. CHCs in a given species can be a mixture of several to more than 100 components of 21–50+ carbon alkanes, alkenes and their branched derivatives that vary in number and position of double bond and methyl branches [1,4]. Such variation makes CHC compositions highly diverse [5], leading to an assortment of mating selection types, and thus informative for reproductive isolation [6].

As a result of species specificity, CHC profiles in distinct species usually display qualitative differences, such as presence or absence of CHC components. However, subtypes of a given species generally vary in the levels of different components [6]. The nature of species specificity makes CHCs an excellent biochemical character to delimit species boundaries [7,8], reveal cryptic species [9–11], and discover new species [12]. Heritable CHCs can be extended to detect intraspecific variation in hydrocarbon phenotypes of some species [13–15]. However, the CHC profiles can be affected by many internal and external factors, such as developmental age, geographic location, and diet [6,16].

Aphids are among the most complex insect species and display multiple intraspecific phenotypic forms [17]. The pea aphid, *Acyrthosiphon pisum* (Harris) is an important pest of many leguminous plants [18,19], and it has been used as a genomic model system for a range of biological studies [17]. Different biotypes usually show various characteristics, including facultative endosymbionts, RNAi efficiency, response to high density, and they can satisfactorily meet the needs of different experiments. As molecular classification is tedious and requires sacrificing sample individuals, there is a growing need for a simple and non-destructive method to delimit the subtypes within *A*. *pisum* species. However, few data are available to address potential CHC profiles in intraspecific delimitation of aphid species and it remains unknown whether species-specific CHC profiles can be used in chemotaxonomic delimitation of biotypes of aphids.

CHCs of *A*. *pisum* are usually extracted by immersing the whole insect into a solvent such as *n*-hexane or dichloromethane and analyzed with GC-MS [20–22]. This method has the risk of extracting internal body lipids and exocrine gland secretions along with the CHCs. A solvent-free and non-destructive solid-phase microextraction (SPME) method with fused-silica fiber coats [23] has been developed for collecting lipids from different matrices [24–27]. SPME seems an ideal approach to study insect CHCs [28]. SPME fibers covered by different coating materials with various polarities are commercially available; however, SPME method and the best fiber have not been developed for studying CHCs in aphids.

In the present study, we examined the efficiency of five different commercial SPME fibers for collecting the *A*. *pisum* CHCs and compared these with the CHCs obtained from hexane extracts. Using the optimized SPME sampling coupled to GC-MS analysis, we explored the polymorphism and plasticity of CHC profiles in *A*. *pisum* with respect to several factors, including developmental stage, wing dimorphism, and host plant. We further investigated the variation of CHC profiles of five different *A*. *pisum* subtypes originally collected from various geographic locations. With qualitative and quantitative analysis of CHC profiles, we conclude that the SPME-based CHC profile can be served as a viable intraspecific taxonomic tool in *A*. *pisum*.

## Materials and methods

### *A*. *pisum* colony

Five discrete geographic morphs of *A*. *pisum* were utilized in this study. Three of them have green body color and two are red. Details of each morph, including body color, original host, collection site, and time are shown in S1 Table. A triplet morph code was assigned for each of the five morphs (GNY, GGS, GYN, RGS or RQH), with the first letter describing body color (G = green; R = red). The five populations were separately reared on either broad bean seedlings (*Vicia faba* L., var. “Jin-nong”) or on clover (*Trifolium repens* L.) in climate chambers at 18°C, ~70% relative humidity (RH), and a 16: 8 h (light: dark) photoperiod. Unless otherwise stated, all aphids used in this study were parthenogenetic, wingless morphs.

### DNA extraction, PCR, and sequencing

Total DNA was isolated from an individual adult using a conventional sodium dodecyl sulfate method [29] and was suspended in 100 μl of nuclease-free water. The purity and integrity were assessed by subjecting 2 μl and 5 μl of total DNA extracts to a NanoDrop 2000c spectrophotometer and 1.2% agarose gel electrophoresis, respectively. We used the primer pair (ApCOI-F: 5’-TTTCAACTAATCATAAAGATATTGG-3’ and ApCOI-R: 5’-TAAACTTCAGGATGTCCAAAGAATCA-3’), which was modified from that designed for Lepidoptera [30], to amplify a 709 bp fragment corresponding to the 5’ region of the *A*. *pisum* mitochondrial COI (mtCOI) gene (GenBank: AB506719). Each PCR reaction mix consisted of 50 μl, containing 5 μl of 10× Ex Taq Buffer, 4 μl of dNTP mixture (2.5 mM each), 2 μl of DNA template, 2 μl of each primer (10 μM), and 0.25 μl of TaKaRa Ex Taq (5 U/μl). The thermocycling profile was set as the following: 94°C for 3 min; 5 cycles of 94°C for 30 s, 45°C for 30 s and 72°C for 50 s followed by 30 cycles of 94°C for 30 s, 51°C for 30 s and 72°C for 50 s; and a final extension of 72°C for 5 min. PCR products were screened on 1% agarose gels and Sanger-based DNA sequencing was performed (Invitrogen, Beijing, China).

### Solvent extraction of CHCs

CHCs were extracted following a 2 min *n*-hexane immersion with two repetitions, as previously described [22]. CHCs of aphids were extracted individually to compare with the SPME method. For quantification of the total amount of CHCs, pooled aphids (~15 mg) were used, and an aliquot of 50 μl of *n*-tetracosane (4 μg/ml in hexane) was added as an internal standard prior to extraction. The hexane extracts were purified by a ~300 mg silica gel (70–230 mesh, Sigma, Louis, MO, USA) mini-column, but for comparison with the SPME method, this purification was not performed. All samples were dried under a gentle nitrogen stream, re-suspended in 50 μl of hexane, and 1 μl aliquot of the solvent was subjected to gas chromatograph (GC) analysis.

### Solid-phase microextraction of CHCs

CHCs of individual aphids were extracted with five different types of SPME fiber assemblies (Supelco, Bellefonte, PA, USA) housed in a manual holder from the same manufacturer. All fibers were sufficiently conditioned by heating them in the injection port of a gas chromatograph (GC) prior to their first use. The temperature and duration of conditioning varied among different stationary phases of fiber coatings, as recommended by the supplier’s instructions: 85 μm carboxen/polydimethylsiloxane (CAR/PDMS) at 300°C for 30 min, 85 μm polyacrylate (PA) at 280°C for 30 min, 65 μm polydimethylsiloxane/divinylbenzene (PDMS/DVB) at 250°C for 30 min, 100 μm PDMS at 250°C for 30 min, and 7 μm PDMS at 320°C for 1 h. SPME sampling was performed as per SPME guidelines [31] to ensure constant conditions for all samples. Live aphids were immobilized on the smaller end of a 1000 μl pipet tip, with which a vacuum pump was equipped to produce negative pressure (see details in S1 Fig). Then the fiber (±1 cm) was rubbed softly and rotated against the abdominal tergum for 30 s. Immediately afterwards, the loaded fibers were inserted into the GC injection port for a 5 min desorption. The fibers were conditioned for 10 min before the next sampling.

### GC-MS analysis

Chemical analyses were performed with a TRACE 1310 gas chromatograph (GC), interfaced to an ISQ single quadruple mass spectrometer (MS) (GC-MS, Thermo Scientific, Waltham, MA, USA), and the system was controlled by the Xcalibur 2.2 software. The GC oven was fit with an HP-5 MS UI capillary column (30 m length × 0.32 mm inner diameter × 0.25 μm film thickness, Agilent Technologies, Santa Clara, CA, USA). Sample injection was performed in splitless mode with helium as the carrier gas at a constant flow of 1 ml min^−1^. Hexane samples (1 μl) were injected by a TriPlus RSH autosampler (Thermo) and SPME samples were manually injected by inserting the fibers into the GC inlet. Specifically for SPME samples, a narrow-bore glass inlet liner (0.75 mm inner diameter) was used to desorb the loaded SPME fibers. The inlet temperature was maintained at 270°C for 65 μm PDMS/DVB, 280°C for 85 μm PA and 100 μm PDMS, 310°C for 85 μm CAR/PDMS, and 320°C for 7 μm PDMS and hexane samples. The GC running of the column oven was programmed from 60°C for 2 min, then ramped at 30°C min^−1^ to 200°C (0 min hold) and ramped at 5°C min^−1^ to 320°C with a 10 min hold. The transfer line was set at 280°C and the mass spectrometer was operated in EI mode with a 70 eV ionization energy. Scanning was recorded from 45 to 650 atomic mass units, at a rate of 5 scans/s.

### Data analysis and statistics

Components of individual peaks were identified by comparing their retention times to those of the *n*-alkane standards (C_7_–C_40_, Sigma, Louis, MO, USA). They were then corroborated by their diagnostic EI ions (m/z = 352, 366, 380, 394, 408, 422, 436, 450, and 464 for C_25_–C_33_ *n*-alkanes, respectively). Each peak area was integrated using a single mass fragment (m/z = 71.0, one of the most intense ions) from the total ion spectrum. The relative proportion (percent area) of each component was computed by dividing individual peak area over the total peak area of all identified components. CHC components for each morph were ranked from high to low according to their relative proportions. For the hexane extracts, quantitative whole amounts of CHCs were calculated by comparing the peak area of each component with that of the internal standard (*IS*, *n*-tetracosane) of known quantity (200 ng).

Nine CHC peaks that occurred regularly were used for statistical analysis. Differences in the percent of individual CHCs found between two groups or among multi-groups were determined with the Student’s *t*-test or one-way ANOVA, followed by the least significant difference test (LSD). CHC variation across groups was investigated with multivariate statistics. To avoid limitations inherent to the analysis of compositional data, the peak area was log-ratio transformed, based on the formula: z_*i*, *j*_ = ln[*Y*_*i*, *j*_/*g*(*Y*_*j*_)], where *Y*_*i*, *j*_ is the area of peak *i* for aphid *j*, *g*(*Y*_*j*_) is the geometric mean of all peaks for aphid *j*, and z_*i*, *j*_ is the standardized area of peak *i* for aphid *j* [32]. The transformed data were then subjected to a principal components analysis (PCA) based on the correlation matrix. Extracted PCs with eigenvalues exceeding 1 were retained, and the results were visualized in score plots. The component matrix that indicates the correlations between each CHC and the PCs was plotted to interpret which CHCs are responsible for separating specific populations. Quantitative differences of CHC profiles among various intraspecific morphs also were determined statistically using multivariate analyses of variance (MANOVA). All statistical analyses were implemented using IBM SPSS Statistics v. 19.0 (SPSS, Inc., Chicago, IL, USA).

## Results

### Variation of mtDNA-COI gene sequence

In order to confirm the identification of *A*. *pisum* species and investigate the genetic variation among different morphs, we sequenced and aligned of the mtCOI gene, which has been recognized as a well-known molecular marker for insect molecular taxonomy. A total of 50 DNA samples (10 for each morph) of PCR products from individual aphids were sequenced. Some of the samples (GGS (2), GYN (1), and RQH (1)) were excluded from the analysis, because of impure signals obtained during the sequencing reaction. Approximately 650 bp sequences were aligned, and a BlastN alignment to the NCBI database revealed that all of the 46 sequences showed a 100% identity to the *A*. *pisum* mtCOI gene.

### Comparison of different SPME fiber coatings

**Fig 1:**
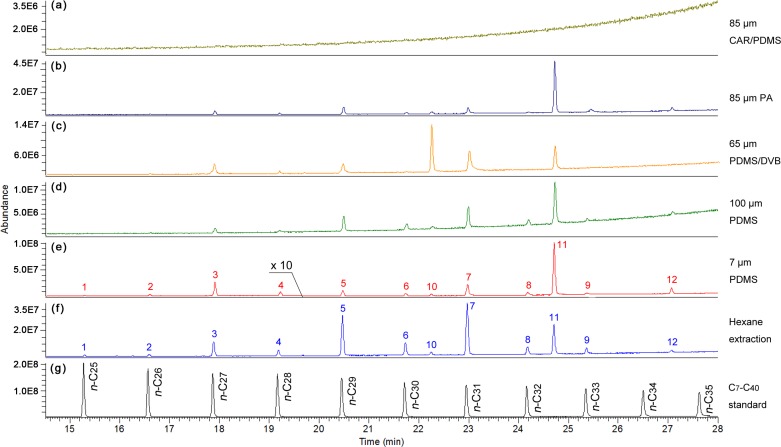
**Representative total ions chromatogram (TIC) of cuticular lipids of *A*. *pisum* extracted by direct SPME fibers (a**–**e) and hexane (f), and of a C**_**7**_–**C**_**40**_ ***n*-alkanes standard (g).** Five different SPME fibers (85 μm CAR/PDMS, 85 μm PA, 65 μm PDMS/DVB, 100 μm PDMS, and 7 μm PDMS), as well as hexane, were tested for the efficiency of CHC detection. Fig 1A–1E were achieved from 2-day old wingless GNY adults. ‘×10’ in Fig 1E indicates that peaks 1–4 are 10 times higher than their actual sizes. Peaks 1–9 in Fig 1E and 1F were tested with the same aphid and identified as C_25_–C_33_ *n*-alkanes, respectively [22]. Peaks 10–12 were identified as aldehydes based on the results of a NIST library (Version 2.0) MS search. Each fiber was run with at least five independent replicates.

To determine an optimum stationary phase of SPME fiber coating for CHC sampling, we compared the chromatograms of cuticular lipids extracted by different types of fibers with different polarities, as well as the organic solvent of *n*-hexane. Of the five types of fibers, the 85 μm CAR/PDMS showed little adsorption of CHCs, with no visual peaks in the chromatogram (Fig 1A). The other coatings, including the 85 μm PA, 65 μm PDMS/DVB, and 100 μm and 7 μm PDMS, resulted in qualitatively similar lipid profiles, each cotaining 12 peaks. However, we found a quantitatively visible difference in the size of CHC peaks among the four coatings (Fig 1B–1E). The signal intensity achieved from the 7 μm PDMS (Fig 1E) was much higher (2.2–10 fold) than those for the other three types of fiber coatings (Fig 1B–1D). For the coating thickness of the PDMS fiber, the 7 μm coating appeared to have a more efficient in adsorption capacity compared to the 100 μm coating. Furthermore, the former possesses a wider range of operating temperatures (220–320°C) compared to the latter (200–280°C), which indicates that the 7 μm coating is more thermostable than the 100 μm coating. Taken together, the fiber coated with 7 μm PDMS was selected as an optimal SPME fiber and was used for all the SPME sampling that followed.

### Reproducibility and validation of SPME-GC analysis

We first investigated the reproducibility of the SPME method by taking repeated samples from the same aphid and computed the coefficient of variation for each of the nine CHC peaks. The coefficients of variation were all less than 30% for the five tested aphid individuals, with averages ranging from 4.1% for *n*-C_31_ to 18.5% for *n*-C_33_ (Table 1). These data indicate that our measurements of CHCs are highly reproducible under the present analytical conditions.

**Table 1:** Coefficient of variation (%) of the measurements for each of the nine CHC components.

Aphid individual	*n*-C_25_	*n*-C_26_	*n*-C_27_	*n*-C_28_	*n*-C_29_	*n*-C_30_	*n*-C_31_	*n*-C_32_	*n*-C_33_
1	5.7	9.2	13.9	9.3	3.0	20.5	3.2	15.2	24.4
2	11.9	6.1	7.2	8.1	5.5	12.5	1.0	12.4	10.3
3	11.8	15.3	16.7	28.2	10.1	12.2	6.1	5.9	26.0
4	15.6	9.5	13.8	17.6	3.4	12.9	4.8	1.9	22.0
5	26.3	17.3	16.1	6.6	2.7	19.9	5.9	7.6	9.7
mean	14.2	11.5	13.5	14.0	4.9	15.6	4.1	8.6	18.5

Wingless adults of the GNY morph were used for direct SPME (7 μm PDMS) sampling, and followed by GC-MS analysis. A coefficient of variation was computed from three sequential measurements for each of five aphid individuals.

**Fig 2:**
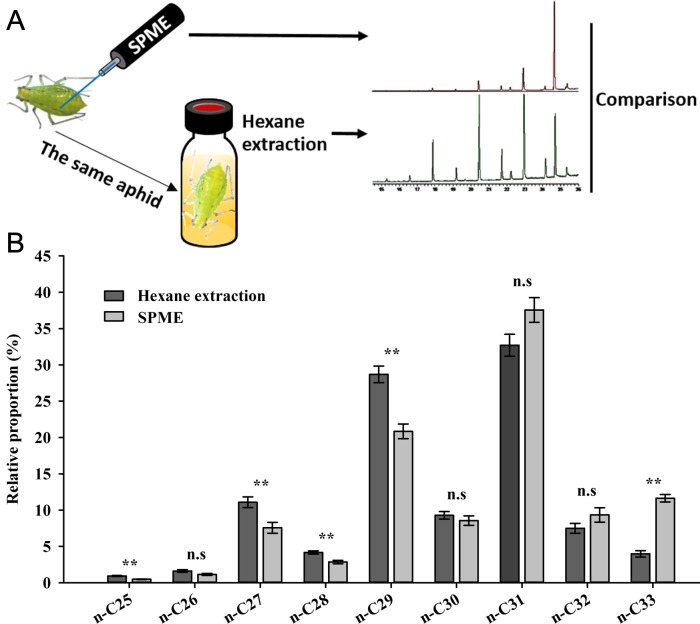
Comparison of CHC profiles of *A*. *pisum* achieved from SPME and hexane extraction. (A) Schematic diagram of experimental procedures for CHCs extraction. CHCs were first extracted with non-destructive SPME, and the same aphid (wingless adult of the GNY morph) was used again for CHCs collection with hexane. (B) Relative proportions of nine CHCs extracted with SPME and hexane. Wingless GNY adults were used. Error bars represent s.e.m of six biological replicates. n.s denotes not significant. * *P* < 0.05, ** *P* < 0.01 (Student’s *t*-test).

Validation of SPME (7 μm PDMS fiber) was performed by comparing the lipid profile with that achieved from hexane extraction (Fig 2A). The two distinct sampling methods showed qualitatively similar results for all five tested morphs: all peaks present in one procedure also were found in the other (Fig 1E and 1F and S2 Fig). The CHC components (peaks 1–9 in either Fig 1E or 1F) have been identified as a series of saturated straight-chain *n*-alkanes (C_25_–C_33_) in previous studies [8,22], and peaks 10–12 were identified as aldehydes, based on the results of a NIST library (Version 2.0) MS search. Nevertheless, the two methods yielded quantitative differences in the relative proportions of some individual components. Compared with the hexane extraction, SPME appeared to reveal lower levels of relatively short chain (≤C_29_) *n*-alkanes (apart from *n*-C_26_) (*n*-C_25_: *P* < 0.001; *n*-C_26_: *P* = 0.067; *n*-C_27_–C_29_: *P* < 0.01), but showed no significant difference in the proportions of relatively long chain (>C_29_) components (apart from *n*-C_33_) (*n*-C_30_: *P* = 0.403; *n*-C_31_: *P* = 0.058; *n*-C_32_: *P* = 0.154; *n*-C_33_: *P* < 0.0001) (Fig 2B).

### Effect of developmental stage, wing dimorphism and host plant on CHC profiles

**Fig 3:**
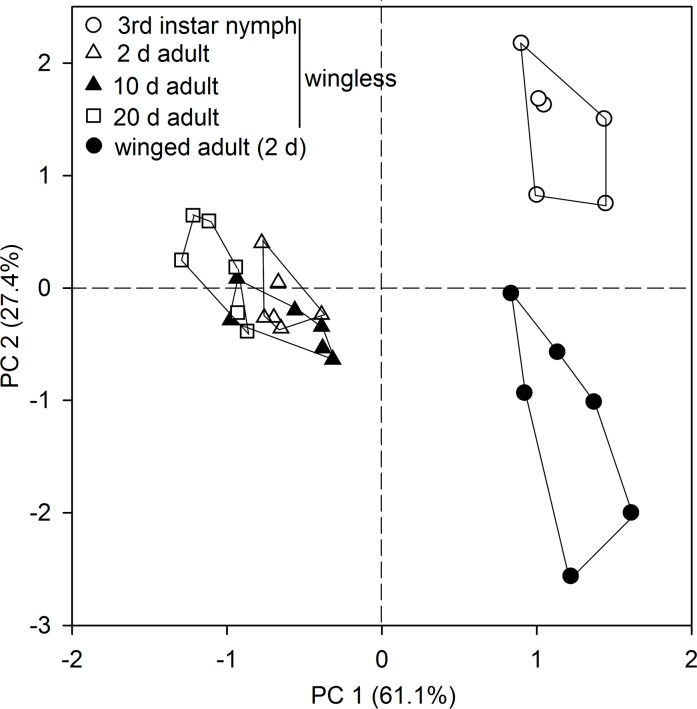
Principal components analysis (PCA) of CHC profiles of the 3rd instar nymphs, wingless and winged adults of *A*. *pisum*. The GNY morph was used for direct SPME sampling with 7 μm PDMS fiber. Shown are score plots of PC 1 versus 2, with the percentage of total variance explained by each axis given in parentheses. Each symbol represents an aphid individual. Data points for each group are enclosed within a line.

We first investigated whether developmental stage and wing dimorphism contribute to CHC variation. Compared with the 3rd instar nymphs, adults showed no distinct differences in the relative abundance of C_25_–C_29_ *n*-alkanes, but they revealed a significant decrease in *n*-C_31_ and an increase in *n*-C_30_, *n*-C_32_, and *n*-C_33_. However, the wingless adults at different ages (2-, 10-, and 20-day old) showed no significant difference in the relative proportion of all components. In addition, winged adult showed a significant increase in the relative proportion of C_25_–C_29_ *n*-alkanes, but a significant decrease in C_30_–C_33_ components (Table 2). The results of MANOVA showed that quantitative CHC profiles were significantly affected by developmental stages and wing dimorphism (Wilks's λ = 0.00008, *F* = 20.763, *P* < 0.0001). A PCA clearly separated aphids by developmental stage (nymph vs. adult) and wing dimorphism (wingless vs. winged), with PC 1 and PC 2 explaining 61.1% and 27.4% of the total variance of the CHC profiles, respectively. Within the wingless adults, aphids of different ages (2-, 10-, and 20-day old) pooled with little variation (Fig 3).

**Table 2:** Percentage of cuticular hydrocarbons at different developmental stages of *Acyrthosiphon pisum*.

	Wingless morph				Winged adult (2 d)	*F* value (df = 4, 25)	*P* value
CHCs	3rd instar nymph	2 d adult	10 d adult	20 d adult			
*n*-C_25_	0.23 ± 0.03 b	0.46 ± 0.03 b	0.48 ± 0.04 b	0.46 ± 0.06 b	1.13 ± 0.32 a	5.276	< 0.01
*n*-C_26_	0.57 ± 0.08 b	0.85 ± 0.05 b	1.15 ± 0.15 b	0.50 ± 0.04 b	2.24 ± 0.41 a	12.214	< 0.0001
*n*-C_27_	8.45 ± 0.74 b	6.60 ± 0.55 bc	7.57 ± 0.75 bc	4.82 ± 0.39 c	20.59 ± 0.94 a	80.634	< 0.0001
*n*-C_28_	3.06 ± 0.30 ab	2.59 ± 0.06 b	2.86 ± 0.23 b	2.45 ± 0.15 b	4.11 ± 0.44 a	5.936	< 0.01
*n*-C_29_	19.71 ± 1.23 b	22.30 ± 0.74 b	20.84 ± 1.02 b	21.55 ± 0.88 b	32.48 ± 1.48 a	22.120	< 0.0001
*n*-C_30_	1.94 ± 0.15 b	8.28 ± 0.48 a	8.57 ± 0.65 a	9.57 ± 0.50 a	3.43 ± 0.17 b	61.440	< 0.0001
*n*-C_31_	60.77 ± 1.57 a	37.44 ± 1.16 b	37.57 ± 1.70 b	35.20 ± 1.06 b	27.84 ± 1.19 c	83.471	< 0.0001
*n*-C_32_	0.52 ± 0.05 b	9.71 ± 1.05 a	9.34 ± 0.98 a	11.80 ± 0.62 a	2.66 ± 0.12 b	48.587	< 0.0001
*n*-C_33_	4.77 ± 0.48 b	11.77 ± 1.62 a	11.63 ± 0.50 a	13.65 ± 0.81 a	5.52 ± 0.53 b	20.051	< 0.0001

The GNY morph was used in this experiment with direct SPME (7 μm PDMS) sampling, and followed by GC-MS analysis. The average (± SE) of six biological replicates is shown for all five groups. Values in each row followed by different letters indicate significant differences (ANOVA, LSD, *P* < 0.05).

**Fig 4:**
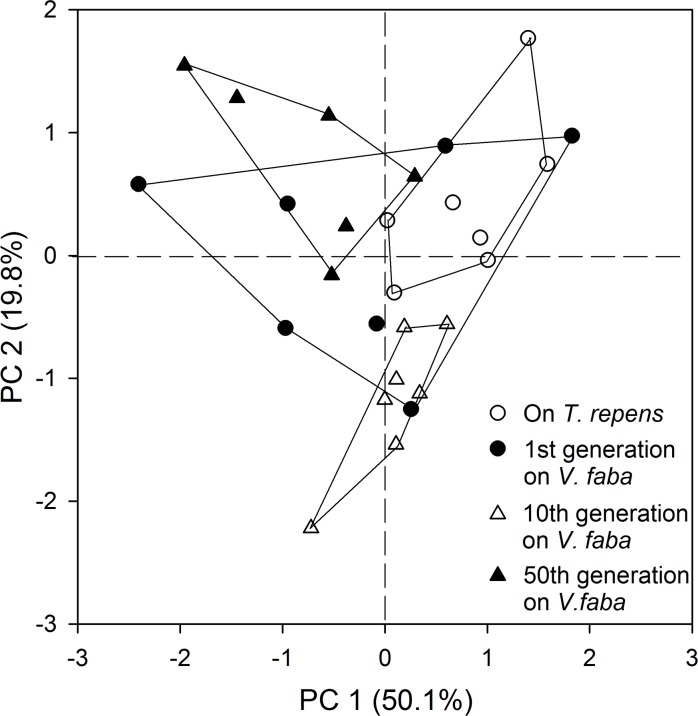
Principal components analysis (PCA) based on CHC composition of *A*. *pisum* during host switching from *T*. *repens* to *V*. *faba*. Wingless adults of the RGS morph were used for SPME sampling with 7 μm PDMS fiber. Shown are score plots of PC 1 versus 2, with the percentage of total variance explained by each axis given in parentheses. Each symbol represents an aphid individual. Data points for each group are enclosed within a line.

The effect of host plant diet on CHC differentiation also was examined by translocating aphids from a well-adapted population on *T*. *renens* to *V*. *faba* for more than 50 generations. The percentages of some components varied significantly at both the 10th and 50th generation, whereas no component revealed significant differences at the first generation (Table 3). A MANOVA of CHC profiles revealed a significant difference due to host plant (Wilks's λ = 0.031, *F* = 3.789, *P* < 0.0001). The principal components plot showed apparent separation of CHC profiles from aphids on *T*. *repens* at both the 10th and 50th, but not the first generation of aphids on *V*. *faba*. The first and second PC explained 50.1% and 19.8% of the total variance of the CHC profiles, respectively (Fig 4).

**Table 3:** Percentage of cuticular hydrocarbons of *Acyrthosiphon pisum* translocated from *Trifolium repens* to *Vicia faba*.

	*T*. *repens*	1st generation on *V*. *faba*		10th generation on *V*. *faba*		50th generation on *V*. *faba*	*F* value (df = 3, 23)	*P* value
*n*-C_25_	0.40 ± 0.02 a		0.40 ± 0.07 a	0.33 ± 0.03 a	0.38 ± 0.01 a		0.822	0.50
*n*-C_26_	1.51 ± 0.07 a		1.15 ± 0.16 ab	1.02 ± 0.06 b	1.04 ± 0.07 b		4.871	< 0.01
*n*-C_27_	11.12 ± 0.69 a		9.15 ± 0.90 ab	8.40 ± 0.37 b	7.65 ± 0.63 b		4.732	< 0.05
*n*-C_28_	7.81 ± 0.63 a		6.69 ± 0.45 a	6.65 ± 0.30 a	7.06 ± 0.28 a		1.475	0.25
*n*-C_29_	37.42 ± 0.73 ab		38.19 ± 1.33 ab	40.80 ± 0.75 a	35.89 ± 0.97 b		4.311	< 0.05
*n*-C_30_	12.56 ± 0.41 ab		13.11 ± 1.46 ab	10.36 ± 0.25 b	14.56 ± 1.02 a		3.521	< 0.05
*n*-C_31_	14.63 ± 0.86 b		17.66 ± 0.81 ab	15.73 ± 0.61 ab	18.28 ± 1.01 a		4.146	< 0.05
*n*-C_32_	3.36 ± 0.14 b		4.78 ± 0.68 ab	3.64 ± 0.09 b	6.37 ± 0.71 a		7.698	< 0.01
*n*-C_33_	11.19 ± 1.11 ab		8.87 ± 0.57 b	13.08 ± 1.11 a	8.77 ± 1.81 b		3.075	< 0.05

The host switching experiment was performed by translocating aphids from *T*. *repens* to *V*. *faba* for various generations. Cuticular hydrocarbon profiles were investigated by direct SPME (7 μm PDMS) sampling and GC-MS analysis. The averages (± SE) of six or seven biological replicates are shown for all of the treatments. Different letters within the same row indicate significant differences (ANOVA, LSD, *P* < 0.05).

### CHC variation among various geographic morphs

**Fig 5:**
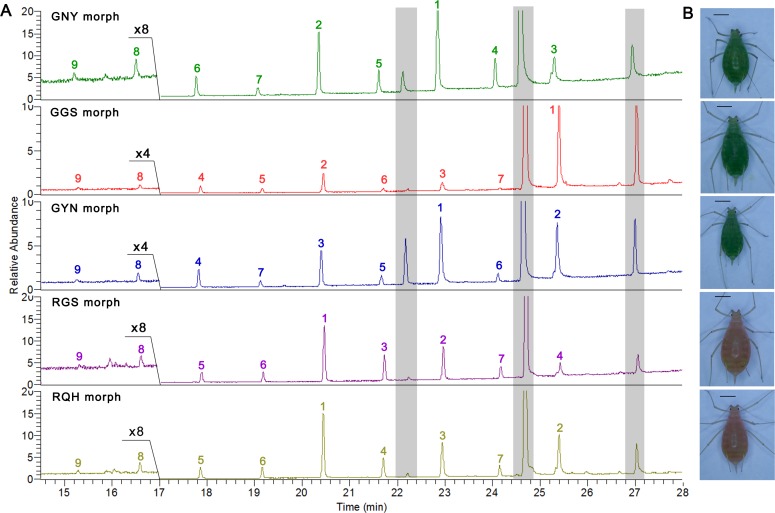
**Representative TIC of cuticular lipids (A) from five geographic morphs of *A*. *pisum* (B).** Wingless adults were used for SPME detection with 7 μm PDMS fiber. Colored numbers above the peaks indicate their rank of peak area for the respective morph (see quantized data in Table 4). ‘×4’ or ‘×8’ in panel (A) indicates that peaks ahead are four or eight times higher than their actual sizes. Peaks with gray shadows indicate the three main cuticular aldehydes. Scale bar in panel (B) is 1 mm.

The intraspecific differences of the CHC profiles among the five geographic morphs were compared based on the rank of relative proportions of the nine major CHC components. The five morphs were strikingly different in the rank numbers for most of the components. These morphs could be distinguished easily based upon one or two principal components. Specifically, *n*-C_33_ was most abundant in the GGS morph. Although *n*-C_31_ also was most abundant in the GNY and GYN morphs, they differed in the second-ranked component (*n*-C_29_ in the GNY morph but *n*-C_33_ in the GYN morph). Similarly, *n*-C_29_ was most abundant in both the RGS and RQH morphs; however, *n*-C_31_ and *n*-C_33_ were the second most abundant compounds in the RGS and RQH morph, respectively (Table 4 and Fig 5).

**Table 4:** Percentage of cuticular hydrocarbons in five intraspecific geographic morphs of *A*. *pisum*.

	GNY morph		GGS morph		GYN morph		RGS morph		RQH morph	
CHCs	% CHC (n = 6)	Rank	% CHC (n = 5)	Rank	% CHC (n = 6)	Rank	% CHC (n = 6)	Rank	% CHC (n = 7)	Rank
*n*-C_25_	0.46 ± 0.03	9	0.52 ± 0.09	9	0.40 ± 0.05	9	0.38 ± 0.01	9	0.29 ± 0.03	9
*n*-C_26_	0.85 ± 0.05	8	1.53 ± 0.23	8	0.80 ± 0.18	8	1.04 ± 0.07	8	0.96 ± 0.12	8
*n*-C_27_	6.60 ± 0.55	6	10.47 ± 1.25	4	8.05 ± 1.17	4	7.65 ± 0.63	5	7.51 ± 0.89	5
*n*-C_28_	2.59 ± 0.06	7	5.59 ± 0.33	5	2.81 ± 0.34	7	7.06 ± 0.28	6	6.09 ± 0.54	6
*n*-C_29_	22.30 ± 0.74	2	24.49 ± 2.12	2	19.36 ± 1.06	3	35.89 ± 0.97	1	34.41 ± 1.35	1
*n*-C_30_	8.28 ± 0.48	5	5.26 ± 1.63	6	5.54 ± 0.44	5	14.56 ± 1.02	3	11.33 ± 0.79	4
*n*-C_31_	37.44 ± 1.16	1	15.65 ± 1.70	3	36.75 ± 1.57	1	18.28 ± 1.01	2	16.50 ± 1.18	3
*n*-C_32_	9.71 ± 1.05	4	2.15 ± 0.74	7	4.42 ± 0.62	6	6.37 ± 0.71	7	4.86 ± 0.63	7
*n*-C_33_	11.77 ± 1.62	3	34.33 ± 4.61	1	21.90 ± 1.83	2	8.77 ± 1.81	4	18.07 ± 2.11	2

Parthenogenetic wingless adults of the five morphs were used for direct SPME (7 μm PDMS) sampling followed by GC-MS analysis.

**Fig 6:**
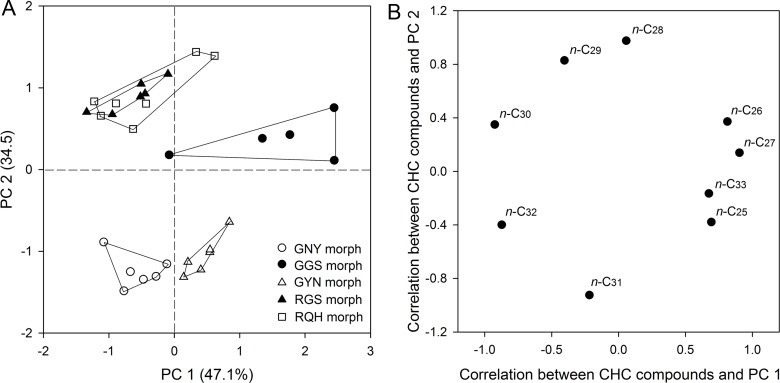
Principal components analysis (PCA) of CHC profiles of five geographic morphs of *A*. *pisum*. Wingless adults of each morph reared on *V*. *faba* were used for SPME sampling (7 μm PDMS). Shown are score plots of PC 1 versus 2, with the percentage of total variance explained by each axis denoted in parentheses. Each symbol represents an aphid individual. Data points for each morph are enclosed within a line. Factor loadings of CHC components on each PC are indicated in panel (B).

A MANOVA showed significant differences in quantitative CHC profiles among geographic morphs (Wilks's λ = 0.002, *F* = 8.335, *P* < 0.0001). A PCA resulted in clear separation of four groups, with some overlap between the RGS and RQH morphs. The first PC accounted for 47.1% of the total variance and separated the three green color morphs (GNY, GGS and GYN) from each other. The second PC which explained 34.5% of the total variance separated the GNY and GYN morphs from the other morphs (Fig 6A). The component matrix indicated that *n*-C_26_ and *n*-C_27 _contributed primarily to group separation along PC 1, and *n*-C_28_ and *n*-C_29_ contributed primarily to separation between these groups along PC 2 (Fig 6B). In addition, the PCA showed that population variation was greater than that caused by host plant for the RGS morph (S3 Fig).

## Discussion

Chemotaxonomy has been recognized as an additional tool for integrated taxonomy for the study of biodiversity. In this study, we have taken advantage of the informative CHC profiles as a novel chemotaxonomy method based on a fiber optimized SPME method coupled to GC-MS analysis. As expected, CHC profiles can be used satisfactorily to classify multiple intraspecific phenotypic forms. More importantly, the five geographic morphs of *A*. *pisum* that showed no sequence variation in the 5’ region of the mtCOI gene could be separated by their CHC profiles. Our findings support the notion that heritable CHC profiles can be a useful biochemical marker for intraspecific delimitation in *A*. *pisum*.

The mtCOI gene, known as a powerful molecular marker for species identification [33], is not suitable for intraspecific taxonomy in *A*. *pisum*. As COI generally provides deeper phylogenetic information than any other mitochondrial gene (e.g., 12S, 16S rDNA, cytochrome *b*) [34,35], we sequenced the COI gene of the five morphs, and did not find any polymorphisms among them. Our data are consistent with a previous study on *A*. *pisum* and several other aphid species (i.e., *Aphis maculatae*, *Hayhurstia atriplicis*, *Myzus persicae* etc.) that exhibited no intraspecific variation of COI sequence among populations collected from various geographic origins in Canada and USA [36], but are slightly different from those in Korea, which showed low variation (average below 0.02% intraspecific pairwise divergence) [37]. Thus, mtCOI works well in molecular identification of reproductively isolated species but is not a reliable marker among populations that continue to exchange genes.

The heritable CHCs provide an excellent approach to chemotaxonomic identification among species, as well as for subtypes within a given species. It is recognized that good taxonomic characters generally fulfill some criteria that make them informative for reproductive isolation and other evolutionary events [38]. As a result of species specificity, the CHC profile shows strikingly qualitative differences among independent species, and considerable data have corroborated this assumption [6,39]. Thus, in the present study, we focused on intraspecific variation. Although CHC profiles within a given species are susceptible to various biological and environmental factors, such factors generally cause differences in CHC quantities rather than alteration in defined components [6,16]. The total amount of CHCs was less useful for discriminating the five colonies, as some of them showed similar CHC levels (S4 Fig). Therefore, we focused more attention on the relative abundance of individual CHC components, traits that were found to be informative and stable during adulthood.

SPME presents several attractive features over the conventional solvent extraction. Most of all, it is a non-destructive method (with no damage or sacrifice of samples), which permits tracking CHC dynamics for valuable specimens. It also allows the study of CHC spatial distribution by rubbing the fiber on a precise location. In addition, SPME is solvent-free, which avoids contamination from internal chemicals and is more suitable for a limited number of specimens including precious museum collections and those with only partial body parts. These advantages prompted our switching to non-lethal SPME from the well-established hexane extraction. We first compared chromatograms achieved from five commercially available SPME fibers and found major quantitative differences among them (Fig 1). As different fibers usually display discrepant polarities designed for various component classes, these differences may reflect different adsorptions of non-polar CHCs, which provided us with further guidelines for fiber selection in CHC study. Although SPME has the shortcoming that absolute quantification of CHCs is not possible, it seems an ideal approach for CHC study, as we only focused on the relative proportions of individual CHCs rather than absolute quantities. We next performed validation of SPME by comparing it to hexane extraction, but we found the two methods resulted in slight quantitative differences (Fig 2). Similarly, previous studies on *Drosophila melanogaster* [25], *Tenebrio molitor* [40], and *Schistocerca gregaria* [41] also revealed this difference in the relative proportions of some CHC components. Unlike quantitative differences, there occurred qualitative differences in CHC profiles of *Melipona marginata* and *Apis mellifera* with the two methods, with more or fewer components detected in one but not the other method [40]. Given that solvent extraction generally involves a whole body wash, rather than a regional investigation in SPME, these differences may be attributed to the non-uniform distribution of cuticular lipids, as recently reported in *D*. *melanogaster* [42]. Moreover, SPME detects only the CHCs present on the outermost layer of epicuticle, while solvent wash also can extract CHCs located in deeper layers of the insect cuticle [43,44]. Therefore, it is more likely that SPME-derived CHCs reflect what is really present on the cuticle surface and should be a preferential consideration for future behavior or other studies.

Several aspects were considered to guide the performance that qualify CHC profiles as a valuable character for intraspecific delimitation in aphids. First of all, adult aphids revealed very low variation in CHC profiles (Fig 3 and Table 2). This stability may be attributed to the slow turnover of CHCs during adulthood in aphids [22] and other insect species [3,45]. One benefit for using adult CHCs is that adulthood allows a broad time range for CHC detection, as it is a terminal stage longer than any nymphal instar. Secondly, the effect of wing dimorphism on the relative proportions of CHCs was also evaluated. We found the winged adults possessed fewer long-chained (>C_29_) CHCs (Table 2) which was positively correlated with a greater desiccation resistance [46,47]. Thus, it is possible that winged aphids may not rely on a high level of desiccation resistance, as they can easily escape from a worse-fitted habitat, such as desiccation stress. The wingless morph was recommended in the present study due to its relatively easy conduct for SPME sampling which can usually be interfered by wings. Finally, we tested whether there were differences in relative proportions of CHC components between aphids feeding on different host plants, as aphids usually involve host switching in the field [18]. Interestingly, we found host switching could lead to the complete differentiation of the aphid CHC profile in only a few generations. Such differentiation highlights the crucial role of host plants in modifying insect CHC profiles, as previously reported in two sympatric beetles, *Altica fragaria* and *Altica viridicyanea* [48]. This suggests that CHC plasticity also could be used for classification of different host biotypes. Given that *A*. *pisum* mostly displays parthenogenetic morphs, and sexual reproductive aphids that lay overwintering eggs are only present in winter [17,49], the CHC profiles of sexual aphids were not investigated here.

The overlap between the RGS and RQH morphs from our PCA was not surprising, though the two morphs could be distinguished by differences in CHC ratios. Such overlap prompts speculation that the two red color morphs may indicate a closer relationship of CHC evolution. It is possible that the two distinct morphs indeed have a similar CHC profile, which makes it difficult to investigate a difference between the groups from our multivariate analysis. Alternatively, the incomplete separation of CHC profiles might reflect on-going colony differentiation, such that the two geographic morphs might belong to an intraspecific colony complex rather than two independent colonies. Such an assumption is based on the relatively close distance (~700 kilometers) between the two respective collection sites and the involvement of colony differentiation in the introduction from one geographic origin to another [18]. Given that CHC composition can well reflect genetic variation inferred from microsatellites among intraspecific colonies of the termite *Reticulitermes santonensis* [15], investigation of microsatellite loci is recommended for further validation of this hypothesis.

Our findings present a novel approach for CHC intraspecific delimitation in hemipterous aphids. However, a full utilization of CHC profiling will require consideration of several aspects regarding sample preparation, SPME sampling, GC analysis, and component identification that will be critical for developing this method in different laboratories. It would be ideal for all sampling and analysis to be completed in one laboratory and by the same operator to ensure all aspects of the method are the same. Conversely, taxonomists should be aware that they may not be able to obtain identical chromatograms to those in this study. The present study merely used the hemipterous *A*. *pisum* as a demonstration of CHC in intraspecific delimitation. While the CHC profile of *A*. *pisum* is rather atypical of insects, as only nine saturated straight-chain *n*-alkanes were detected, the methodology likely will work equally well for other complex CHC profiles. All in all, taxonomists should be aware of the limitations listed above when taking advantage of CHC profiles for taxonomic studies in other insect species.

Development of cloud-based databases and a retrieval platform will provide universal guidelines for such concerns and thus facilitate the standard utilization of CHC-based chemotaxonomy. A promising application is the development of a database for a limited number of quarantine pests of various geographic populations, which will provide information on the imported routes of these pests. The CHC profiles reported here may provide a complementary approach to the well-established methods of insect taxonomy based on morphology and genetics. A combination of CHC profile and conventional tools should be encouraged to drive the development of integrative insect taxonomy. Clearly, the establishment of SPME-based CHC profiling is just in its infancy and more extensive studies will be necessary to supplement and optimize the utilization of CHC phenotypes in the field of insect chemotaxonomy.

## Supporting information

S1 Table Details of the five geographic morphs of *A*. *pisum* utilized in this study. (PDF)

S1 Fig Device assembly for SPME sampling of cuticular lipids on *A*. *pisum*. (PDF)

S2 Fig Representative total ions chromatogram (TIC) of cuticular lipids of *A*. *pisum* from SPME and hexane extraction. (PDF)

S3 Fig Principal components analysis (PCA) of CHC composition of five geographic morphs of *A*. *pisum* on host plants *V*. *faba* and *T*. *repens*. (PDF)

S4 Fig Total amount of CHCs in five intraspecific morphs of *A*. *pisum*. (PDF)

S1 Appendix DNA sequences of the COI gene from five geographic morphs of *A*. *pisum*. (DOCX)

S2 Appendix Relative proportions of individual CHCs from various intraspecific morphs of *A*. *pisum*. (XLSX)
