# Supplementary material for: Solid-phase microextraction-based cuticular hydrocarbon profiling for intraspecific delimitation in Acyrthosiphon pisum
Source: PLoS One. 2017 Aug 31;12(8):e0184243. doi: 10.1371/journal.pone.0184243 (PMC5578635; doi:10.1371/journal.pone.0184243)
Supplement: S1 Table — (PDF) [file pone.0184243.s001.pdf]

S1 Table. Details of the five geographic morphs of *Acyrthosiphon pisum* utilized in this study.

Morph code	Body color	Collection site	Collection Time	Original host
GNY	Green	Freeville, New York, USA	2009	*Medicago sativa*
GGS	Green	Lanzhou, Gansu, China	Aug. 2014	*Medicago sativa*
GYN	Green	Yuxi, Yunnan, China	Mar. 2010	*Vicia faba*
RGS	Red	Lanzhou, Gansu, China	Apr. 2013	*Medicago sativa*
RQH	Red	Delhi, Qinghai, China	Aug. 2014	*Medicago sativa*
