# Supplementary figures and images for: Solid-phase microextraction-based cuticular hydrocarbon profiling for intraspecific delimitation in Acyrthosiphon pisum
Source: PLoS One. 2017 Aug 31;12(8):e0184243. doi: 10.1371/journal.pone.0184243 (PMC5578635; doi:10.1371/journal.pone.0184243)

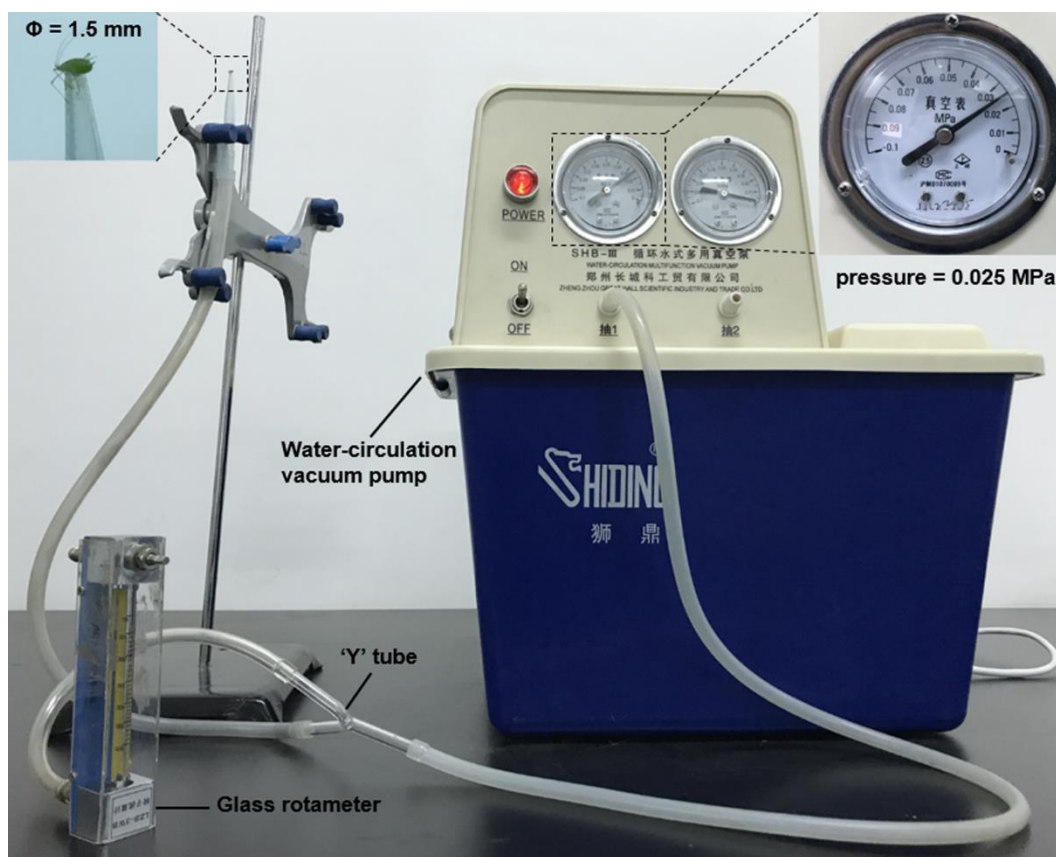

**S1 Fig. Device assembly for SPME sampling of cuticular lipids on pea aphids.**

Supplement: S1 Fig — (PDF) [file pone.0184243.s002.pdf]
