# Supplementary material for: Solid-phase microextraction-based cuticular hydrocarbon profiling for intraspecific delimitation in Acyrthosiphon pisum
Source: PLoS One. 2017 Aug 31;12(8):e0184243. doi: 10.1371/journal.pone.0184243 (PMC5578635; doi:10.1371/journal.pone.0184243)
Supplement: S2 Fig — (PDF) [file pone.0184243.s003.pdf]

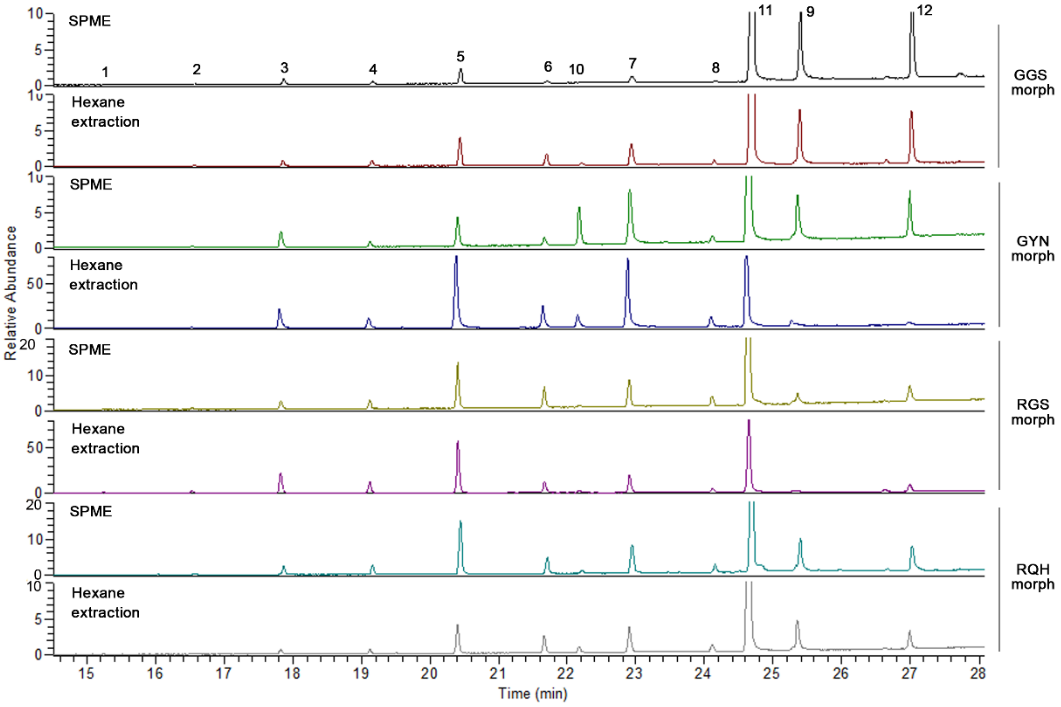

S2 Fig. Representative total ions chromatogram (TIC) of cuticular lipids of *A. pisum* from SPME and hexane extraction. CHCs were first extracted with non-destructive SPME, and the same aphid (wingless adult of the GGS, GYN, RGS or RQH morph) was used again for CHCs collection with hexane. Peaks 1–9 have been identified as C_{25}–C_{33} *n*-alkanes, respectively. Peaks 10–12 were identified as aldehydes based on the results of a NIST library (Version 2.0) MS search.
