# Supplementary material for: Solid-phase microextraction-based cuticular hydrocarbon profiling for intraspecific delimitation in Acyrthosiphon pisum
Source: PLoS One. 2017 Aug 31;12(8):e0184243. doi: 10.1371/journal.pone.0184243 (PMC5578635; doi:10.1371/journal.pone.0184243)
Supplement: S3 Fig — (PDF) [file pone.0184243.s004.pdf]

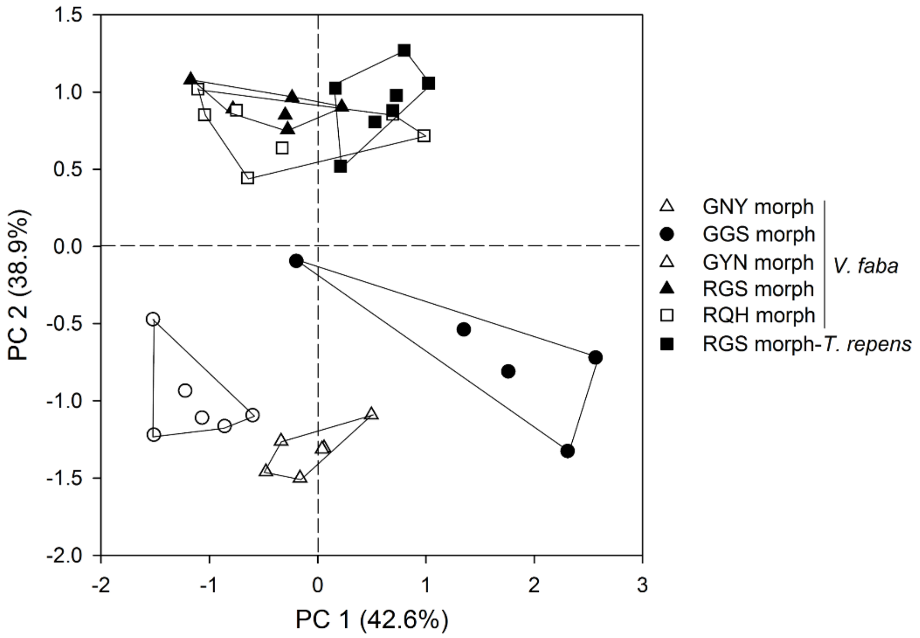

S3 Fig. Principal components analysis (PCA) of CHC composition of five geographic morphs of *A. pisum* on host plants *V. faba* and *T. repens*. Wingless adults of each morph reared on *V. faba* or *T. repens* were used for SPME sampling (7 µm PDMS). Shown are score plots of PC 1 versus 2 with percentages of total variance denoted in parentheses. Each symbol represents an aphid individual. Data points for each group are enclosed with a line.
