# Supplementary material for: Solid-phase microextraction-based cuticular hydrocarbon profiling for intraspecific delimitation in Acyrthosiphon pisum
Source: PLoS One. 2017 Aug 31;12(8):e0184243. doi: 10.1371/journal.pone.0184243 (PMC5578635; doi:10.1371/journal.pone.0184243)
Supplement: S4 Fig — (PDF) [file pone.0184243.s005.pdf]

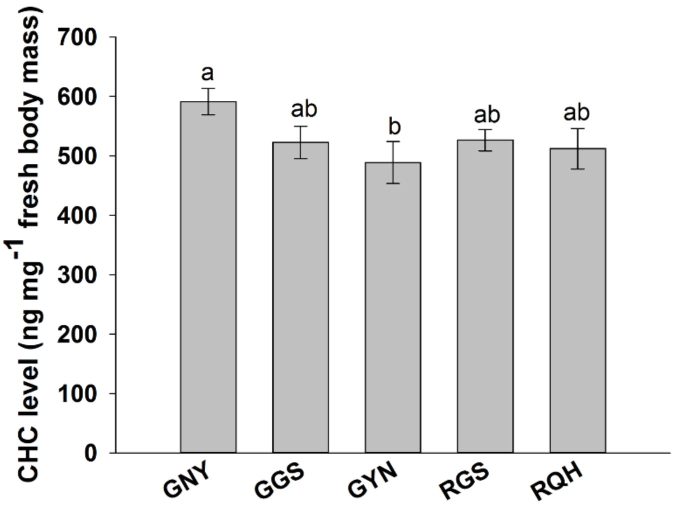

S4 Fig. Total amount of CHCs in five intraspecific morphs of *A. pisum*. Cuticular hydrocarbons (nanogram per milligram of fresh body mass ± SE) of 2-day old adults were extracted by hexane and quantified by GC-MS. Error bars represent SE of five biological replicates. Different letters indicate significant differences among different intraspecific morphs (ANOVA, LSD, $P < 0.05$).
