# Supplementary material for: Solid-phase microextraction-based cuticular hydrocarbon profiling for intraspecific delimitation in Acyrthosiphon pisum
Source: PLoS One. 2017 Aug 31;12(8):e0184243. doi: 10.1371/journal.pone.0184243 (PMC5578635; doi:10.1371/journal.pone.0184243)
Supplement: S1 Appendix — (DOCX) [file pone.0184243.s006.docx]

**GNY-1:**

ATCTTTATTTGGTaTTTGATCAGGTATAATTGGATCTTCACTTAGAATTCTAATTCGTTTAGAATTAAGTCAAATTAATT

CTATTATTAACAATAATCAATTATATAATGTAATTGTTACAATTCATGCTTTTATTATAATTTTTTTTATAACTATACCA

ATTGTAATTGGTGGATTTGGAAATTGATTAATTCCTATAATAATAGGATGTCCTGATATATCATTTCCTCGCTTAAATAA

TATTAGATTTTGATTATTACCTCCTTCATTAATAATAATAATTTGCAGTTTCTTAATTAATAATGGAACAGGAACAGGAT

GAACTATTTATCCACCTTTATCAAATAATATTGCACATAATAACATTTCAGTTGATTTAACTATTTTTTCTTTACACCTA

GCAGGAATTTCATCAATTTTAGGAGCAATTAATTTTATTTGTACAATTCTTAATATAATACCTAATAACATAAAATTAAA

TCAAATTCCACTTTTCCCTTGATCAATTTTAATTACAGCTATCTTATTAATTTTATCTTTACCAGTTTTAGCTGGTGCTA

TTACAATATTATTAACTGATCGAAACTTAAATACATCATTTTTTGATCCAGCAGGAGGAGGAGATCCTATTTTATACCAA

CATTTATTTTGATTCTTTGGCACC

**GNY-2:**

CTCTCCCTTGGTaTTTGATCAGGTATAATTGGATCTTCACTTAGAATTCTAATTCGTTTAGAATTAAGTCAAATTAATTC

TATTATTAACAATAATCAATTATATAATGTAATTGTTACAATTCATGCTTTTATTATAATTTTTTTTATAACTATACCAA

TTGTAATTGGTGGATTTGGAAATTGATTAATTCCTATAATAATAGGATGTCCTGATATATCATTTCCTCGCTTAAATAAT

ATTAGATTTTGATTATTACCTCCTTCATTAATAATAATAATTTGCAGTTTCTTAATTAATAATGGAACAGGAACAGGATG

AACTATTTATCCACCTTTATCAAATAATATTGCACATAATAACATTTCAGTTGATTTAACTATTTTTTCTTTACACCTAG

CAGGAATTTCATCAATTTTAGGAGCAATTAATTTTATTTGTACAATTCTTAATATAATACCTAATAACATAAAATTAAAT

CAAATTCCACTTTTCCCTTGATCAATTTTAATTACAGCTATCTTATTAATTTTATCTTTACCAGTTTTAGCTGGTGCTAT

TACAATATTATTAACTGATCGAAACTTAAATACATCATTTTTTGATCCAGCAGGAGGAGGAGATCCTATTTTATACCAAC

ATTTATTTTGATTCTTTGGCACC

**GNY-3:**

ATCTCTTTGGTaTTTGATCAGGTATAATTGGATCTTCACTTAGAATTCTAATTCGTTTAGAATTAAGTCAAATTAATTCT

ATTATTAACAATAATCAATTATATAATGTAATTGTTACAATTCATGCTTTTATTATAATTTTTTTTATAACTATACCAAT

TGTAATTGGTGGATTTGGAAATTGATTAATTCCTATAATAATAGGATGTCCTGATATATCATTTCCTCGCTTAAATAATA

TTAGATTTTGATTATTACCTCCTTCATTAATAATAATAATTTGCAGTTTCTTAATTAATAATGGAACAGGAACAGGATGA

ACTATTTATCCACCTTTATCAAATAATATTGCACATAATAACATTTCAGTTGATTTAACTATTTTTTCTTTACACCTAGC

AGGAATTTCATCAATTTTAGGAGCAATTAATTTTATTTGTACAATTCTTAATATAATACCTAATAACATAAAATTAAATC

AAATTCCACTTTTCCCTTGATCAATTTTAATTACAGCTATCTTATTAATTTTATCTTTACCAGTTTTAGCTGGTGCTATT

ACAATATTATTAACTGATCGAAACTTAAATACATCATTTTTTGATCCAGCAGGAGGAGGAGATCCTATTTTATACCAACA

TTTATTTTGATTCTTTGGCCCGGG

**GNY-4:**

CTACTTTATTGGTaTTTGATCAGGTATAATTGGATCTTCACTTAGAATTCTAATTCGTTTAGAATTAAGTCAAATTAATT

CTATTATTAACAATAATCAATTATATAATGTAATTGTTACAATTCATGCTTTTATTATAATTTTTTTTATAACTATACCA

ATTGTAATTGGTGGATTTGGAAATTGATTAATTCCTATAATAATAGGATGTCCTGATATATCATTTCCTCGCTTAAATAA

TATTAGATTTTGATTATTACCTCCTTCATTAATAATAATAATTTGCAGTTTCTTAATTAATAATGGAACAGGAACAGGAT

GAACTATTTATCCACCTTTATCAAATAATATTGCACATAATAACATTTCAGTTGATTTAACTATTTTTTCTTTACACCTA

GCAGGAATTTCATCAATTTTAGGAGCAATTAATTTTATTTGTACAATTCTTAATATAATACCTAATAACATAAAATTAAA

TCAAATTCCACTTTTCCCTTGATCAATTTTAATTACAGCTATCTTATTAATTTTATCTTTACCAGTTTTAGCTGGTGCTA

TTACAATATTATTAACTGATCGAAACTTAAATACATCATTTTTTGATCCAGCAGGAGGAGGAGATCCTATTTTATACCAA

CATTTATTTTGATTCTTTGGCACC

**GNY-5:**

ATTGGTaTTTGATCAGGTATAATTGGATCTTCACTTAGAATTCTAATTCGTTTAGAATTAAGTCAAATTAATTCTATTAT

TAACAATAATCAATTATATAATGTAATTGTTACAATTCATGCTTTTATTATAATTTTTTTTATAACTATACCAATTGTAA

TTGGTGGATTTGGAAATTGATTAATTCCTATAATAATAGGATGTCCTGATATATCATTTCCTCGCTTAAATAATATTAGA

TTTTGATTATTACCTCCTTCATTAATAATAATAATTTGCAGTTTCTTAATTAATAATGGAACAGGAACAGGATGAACTAT

TTATCCACCTTTATCAAATAATATTGCACATAATAACATTTCAGTTGATTTAACTATTTTTTCTTTACACCTAGCAGGAA

TTTCATCAATTTTAGGAGCAATTAATTTTATTTGTACAATTCTTAATATAATACCTAATAACATAAAATTAAATCAAATT

CCACTTTTCCCTTGATCAATTTTAATTACAGCTATCTTATTAATTTTATCTTTACCAGTTTTAGCTGGTGCTATTACAAT

ATTATTAACTGATCGAAACTTAAATACATCATTTTTTGATCCAGCAGGAGGAGGAGATCCTATTTTATACCAACATTTAT

TTTGATTCTTTGGCACC

**GNY-6:**

ATTGGTaTTTGATCAGGTATAATTGGATCTTCACTTAGAATTCTAATTCGTTTAGAATTAAGTCAAATTAATTCTATTAT

TAACAATAATCAATTATATAATGTAATTGTTACAATTCATGCTTTTATTATAATTTTTTTTATAACTATACCAATTGTAA

TTGGTGGATTTGGAAATTGATTAATTCCTATAATAATAGGATGTCCTGATATATCATTTCCTCGCTTAAATAATATTAGA

TTTTGATTATTACCTCCTTCATTAATAATAATAATTTGCAGTTTCTTAATTAATAATGGAACAGGAACAGGATGAACTAT

TTATCCACCTTTATCAAATAATATTGCACATAATAACATTTCAGTTGATTTAACTATTTTTTCTTTACACCTAGCAGGAA

TTTCATCAATTTTAGGAGCAATTAATTTTATTTGTACAATTCTTAATATAATACCTAATAACATAAAATTAAATCAAATT

CCACTTTTCCCTTGATCAATTTTAATTACAGCTATCTTATTAATTTTATCTTTACCAGTTTTAGCTGGTGCTATTACAAT

ATTATTAACTGATCGAAACTTAAATACATCATTTTTTGATCCAGCAGGAGGAGGAGATCCTATTTTATACCAACATTTAT

TTTGATTCTTTGGCA

**GNY-7:**

CTTTTTGGTaTTTGATCAGGTATAATTGGATCTTCACTTAGAATTCTAATTCGTTTAGAATTAAGTCAAATTAATTCTAT

TATTAACAATAATCAATTATATAATGTAATTGTTACAATTCATGCTTTTATTATAATTTTTTTTATAACTATACCAATTG

TAATTGGTGGATTTGGAAATTGATTAATTCCTATAATAATAGGATGTCCTGATATATCATTTCCTCGCTTAAATAATATT

AGATTTTGATTATTACCTCCTTCATTAATAATAATAATTTGCAGTTTCTTAATTAATAATGGAACAGGAACAGGATGAAC

TATTTATCCACCTTTATCAAATAATATTGCACATAATAACATTTCAGTTGATTTAACTATTTTTTCTTTACACCTAGCAG

GAATTTCATCAATTTTAGGAGCAATTAATTTTATTTGTACAATTCTTAATATAATACCTAATAACATAAAATTAAATCAA

ATTCCACTTTTCCCTTGATCAATTTTAATTACAGCTATCTTATTAATTTTATCTTTACCAGTTTTAGCTGGTGCTATTAC

AATATTATTAACTGATCGAAACTTAAATACATCATTTTTTGATCCAGCAGGAGGAGGAGATCCTATTTTATACCAACATT

TATTTTGATTCTTTGGAACC

**GNY-8:**

CTACTTTATTGGTaTTTGATCAGGTATAATTGGATCTTCACTTAGAATTCTAATTCGTTTAGAATTAAGTCAAATTAATT

CTATTATTAACAATAATCAATTATATAATGTAATTGTTACAATTCATGCTTTTATTATAATTTTTTTTATAACTATACCA

ATTGTAATTGGTGGATTTGGAAATTGATTAATTCCTATAATAATAGGATGTCCTGATATATCATTTCCTCGCTTAAATAA

TATTAGATTTTGATTATTACCTCCTTCATTAATAATAATAATTTGCAGTTTCTTAATTAATAATGGAACAGGAACAGGAT

GAACTATTTATCCACCTTTATCAAATAATATTGCACATAATAACATTTCAGTTGATTTAACTATTTTTTCTTTACACCTA

GCAGGAATTTCATCAATTTTAGGAGCAATTAATTTTATTTGTACAATTCTTAATATAATACCTAATAACATAAAATTAAA

TCAAATTCCACTTTTCCCTTGATCAATTTTAATTACAGCTATCTTATTAATTTTATCTTTACCAGTTTTAGCTGGTGCTA

TTACAATATTATTAACTGATCGAAACTTAAATACATCATTTTTTGATCCAGCAGGAGGAGGAGATCCTATTTTATACCAA

CATTTATTTTGATTCTTTGGACACC

**GNY-9:**

ATCTTTTATTTGGTaTTTGATCAGGTATAATTGGATCTTCACTTAGAATTCTAATTCGTTTAGAATTAAGTCAAATTAAT

TCTATTATTAACAATAATCAATTATATAATGTAATTGTTACAATTCATGCTTTTATTATAATTTTTTTTATAACTATACC

AATTGTAATTGGTGGATTTGGAAATTGATTAATTCCTATAATAATAGGATGTCCTGATATATCATTTCCTCGCTTAAATA

ATATTAGATTTTGATTATTACCTCCTTCATTAATAATAATAATTTGCAGTTTCTTAATTAATAATGGAACAGGAACAGGA

TGAACTATTTATCCACCTTTATCAAATAATATTGCACATAATAACATTTCAGTTGATTTAACTATTTTTTCTTTACACCT

AGCAGGAATTTCATCAATTTTAGGAGCAATTAATTTTATTTGTACAATTCTTAATATAATACCTAATAACATAAAATTAA

ATCAAATTCCACTTTTCCCTTGATCAATTTTAATTACAGCTATCTTATTAATTTTATCTTTACCAGTTTTAGCTGGTGCT

ATTACAATATTATTAACTGATCGAAACTTAAATACATCATTTTTTGATCCAGCAGGAGGAGGAGATCCTATTTTATACCA

ACATTTATTTTGATTCTTTGGACTC

**GNY-10:**

ATTTTATTTGGTaTTTGATCAGGTATAATTGGATCTTCACTTAGAATTCTAATTCGTTTAGAATTAAGTCAAATTAATTC

TATTATTAACAATAATCAATTATATAATGTAATTGTTACAATTCATGCTTTTATTATAATTTTTTTTATAACTATACCAA

TTGTAATTGGTGGATTTGGAAATTGATTAATTCCTATAATAATAGGATGTCCTGATATATCATTTCCTCGCTTAAATAAT

ATTAGATTTTGATTATTACCTCCTTCATTAATAATAATAATTTGCAGTTTCTTAATTAATAATGGAACAGGAACAGGATG

AACTATTTATCCACCTTTATCAAATAATATTGCACATAATAACATTTCAGTTGATTTAACTATTTTTTCTTTACACCTAG

CAGGAATTTCATCAATTTTAGGAGCAATTAATTTTATTTGTACAATTCTTAATATAATACCTAATAACATAAAATTAAAT

CAAATTCCACTTTTCCCTTGATCAATTTTAATTACAGCTATCTTATTAATTTTATCTTTACCAGTTTTAGCTGGTGCTAT

TACAATATTATTAACTGATCGAAACTTAAATACATCATTTTTTGATCCAGCAGGAGGAGGAGATCCTATTTTATACCAAC

ATTTATTTTGATTCTTTGGACTC

**GGS-1:**

CTTTGGTaTTTGATCAGGTATAATTGGATCTTCACTTAGAATTCTAATTCGTTTAGAATTAAGTCAAATTAATTCTATTA

TTAACAATAATCAATTATATAATGTAATTGTTACAATTCATGCTTTTATTATAATTTTTTTTATAACTATACCAATTGTA

ATTGGTGGATTTGGAAATTGATTAATTCCTATAATAATAGGATGTCCTGATATATCATTTCCTCGCTTAAATAATATTAG

ATTTTGATTATTACCTCCTTCATTAATAATAATAATTTGCAGTTTCTTAATTAATAATGGAACAGGAACAGGATGAACTA

TTTATCCACCTTTATCAAATAATATTGCACATAATAACATTTCAGTTGATTTAACTATTTTTTCTTTACACCTAGCAGGA

ATTTCATCAATTTTAGGAGCAATTAATTTTATTTGTACAATTCTTAATATAATACCTAATAACATAAAATTAAATCAAAT

TCCACTTTTCCCTTGATCAATTTTAATTACAGCTATCTTATTAATTTTATCTTTACCAGTTTTAGCTGGTGCTATTACAA

TATTATTAACTGATCGAAACTTAAATACATCATTTTTTGATCCAGCAGGAGGAGGAGATCCTATTTTATACCAACATTTA

TTTTGATTCTTTGGACA

**GGS-2:**

CATTTGGTaTTTGATCAGGTATAATTGGATCTTCACTTAGAATTCTAATTCGTTTAGAATTAAGTCAAATTAATTCTATT

ATTAACAATAATCAATTATATAATGTAATTGTTACAATTCATGCTTTTATTATAATTTTTTTTATAACTATACCAATTGT

AATTGGTGGATTTGGAAATTGATTAATTCCTATAATAATAGGATGTCCTGATATATCATTTCCTCGCTTAAATAATATTA

GATTTTGATTATTACCTCCTTCATTAATAATAATAATTTGCAGTTTCTTAATTAATAATGGAACAGGAACAGGATGAACT

ATTTATCCACCTTTATCAAATAATATTGCACATAATAACATTTCAGTTGATTTAACTATTTTTTCTTTACACCTAGCAGG

AATTTCATCAATTTTAGGAGCAATTAATTTTATTTGTACAATTCTTAATATAATACCTAATAACATAAAATTAAATCAAA

TTCCACTTTTCCCTTGATCAATTTTAATTACAGCTATCTTATTAATTTTATCTTTACCAGTTTTAGCTGGTGCTATTACA

ATATTATTAACTGATCGAAACTTAAATACATCATTTTTTGATCCAGCAGGAGGAGGAGATCCTATTTTATACCAACATTT

ATTTTGATTCTTTGGACACC

**GGS-3:**

CTTTATTTGGTaTTTGATCAGGTATAATTGGATCTTCACTTAGAATTCTAATTCGTTTAGAATTAAGTCAAATTAATTCT

ATTATTAACAATAATCAATTATATAATGTAATTGTTACAATTCATGCTTTTATTATAATTTTTTTTATAACTATACCAAT

TGTAATTGGTGGATTTGGAAATTGATTAATTCCTATAATAATAGGATGTCCTGATATATCATTTCCTCGCTTAAATAATA

TTAGATTTTGATTATTACCTCCTTCATTAATAATAATAATTTGCAGTTTCTTAATTAATAATGGAACAGGAACAGGATGA

ACTATTTATCCACCTTTATCAAATAATATTGCACATAATAACATTTCAGTTGATTTAACTATTTTTTCTTTACACCTAGC

AGGAATTTCATCAATTTTAGGAGCAATTAATTTTATTTGTACAATTCTTAATATAATACCTAATAACATAAAATTAAATC

AAATTCCACTTTTCCCTTGATCAATTTTAATTACAGCTATCTTATTAATTTTATCTTTACCAGTTTTAGCTGGTGCTATT

ACAATATTATTAACTGATCGAAACTTAAATACATCATTTTTTGATCCAGCAGGAGGAGGAGATCCTATTTTATACCAACA

TTTATTTTGATTCTTTGGAAC

**GGS-4:**

ACTTATTGGTaTTTGATCAGGTATAATTGGATCTTCACTTAGAATTCTAATTCGTTTAGAATTAAGTCAAATTAATTCTA

TTATTAACAATAATCAATTATATAATGTAATTGTTACAATTCATGCTTTTATTATAATTTTTTTTATAACTATACCAATT

GTAATTGGTGGATTTGGAAATTGATTAATTCCTATAATAATAGGATGTCCTGATATATCATTTCCTCGCTTAAATAATAT

TAGATTTTGATTATTACCTCCTTCATTAATAATAATAATTTGCAGTTTCTTAATTAATAATGGAACAGGAACAGGATGAA

CTATTTATCCACCTTTATCAAATAATATTGCACATAATAACATTTCAGTTGATTTAACTATTTTTTCTTTACACCTAGCA

GGAATTTCATCAATTTTAGGAGCAATTAATTTTATTTGTACAATTCTTAATATAATACCTAATAACATAAAATTAAATCA

AATTCCACTTTTCCCTTGATCAATTTTAATTACAGCTATCTTATTAATTTTATCTTTACCAGTTTTAGCTGGTGCTATTA

CAATATTATTAACTGATCGAAACTTAAATACATCATTTTTTGATCCAGCAGGAGGAGGAGATCCTATTTTATACCAACAT

TTATTTTGATTCTTTGGAACTGGGG

**GGS-7:**

CTATTTGGTaTTTGATCAGGTATAATTGGATCTTCACTTAGAATTCTAATTCGTTTAGAATTAAGTCAAATTAATTCTAT

TATTAACAATAATCAATTATATAATGTAATTGTTACAATTCATGCTTTTATTATAATTTTTTTTATAACTATACCAATTG

TAATTGGTGGATTTGGAAATTGATTAATTCCTATAATAATAGGATGTCCTGATATATCATTTCCTCGCTTAAATAATATT

AGATTTTGATTATTACCTCCTTCATTAATAATAATAATTTGCAGTTTCTTAATTAATAATGGAACAGGAACAGGATGAAC

TATTTATCCACCTTTATCAAATAATATTGCACATAATAACATTTCAGTTGATTTAACTATTTTTTCTTTACACCTAGCAG

GAATTTCATCAATTTTAGGAGCAATTAATTTTATTTGTACAATTCTTAATATAATACCTAATAACATAAAATTAAATCAA

ATTCCACTTTTCCCTTGATCAATTTTAATTACAGCTATCTTATTAATTTTATCTTTACCAGTTTTAGCTGGTGCTATTAC

AATATTATTAACTGATCGAAACTTAAATACATCATTTTTTGATCCAGCAGGAGGAGGAGATCCTATTTTATACCAACATT

TATTTTGATTCTTTGGCACTGGAAAAAATTTTAA

**GGS-8:**

CTGGTaTTTGATCAGGTATAATTGGATCTTCACTTAGAATTCTAATTCGTTTAGAATTAAGTCAAATTAATTCTATTATT

AACAATAATCAATTATATAATGTAATTGTTACAATTCATGCTTTTATTATAATTTTTTTTATAACTATACCAATTGTAAT

TGGTGGATTTGGAAATTGATTAATTCCTATAATAATAGGATGTCCTGATATATCATTTCCTCGCTTAAATAATATTAGAT

TTTGATTATTACCTCCTTCATTAATAATAATAATTTGCAGTTTCTTAATTAATAATGGAACAGGAACAGGATGAACTATT

TATCCACCTTTATCAAATAATATTGCACATAATAACATTTCAGTTGATTTAACTATTTTTTCTTTACACCTAGCAGGAAT

TTCATCAATTTTAGGAGCAATTAATTTTATTTGTACAATTCTTAATATAATACCTAATAACATAAAATTAAATCAAATTC

CACTTTTCCCTTGATCAATTTTAATTACAGCTATCTTATTAATTTTATCTTTACCAGTTTTAGCTGGTGCTATTACAATA

TTATTAACTGATCGAAACTTAAATACATCATTTTTTGATCCAGCAGGAGGAGGAGATCCTATTTTATACCAACATTTATT

TTGATTCTTTGGACACCGGG

**GGS-9:**

CTGGTaTTTGATCAGGTATAATTGGATCTTCACTTAGAATTCTAATTCGTTTAGAATTAAGTCAAATTAATTCTATTATT

AACAATAATCAATTATATAATGTAATTGTTACAATTCATGCTTTTATTATAATTTTTTTTATAACTATACCAATTGTAAT

TGGTGGATTTGGAAATTGATTAATTCCTATAATAATAGGATGTCCTGATATATCATTTCCTCGCTTAAATAATATTAGAT

TTTGATTATTACCTCCTTCATTAATAATAATAATTTGCAGTTTCTTAATTAATAATGGAACAGGAACAGGATGAACTATT

TATCCACCTTTATCAAATAATATTGCACATAATAACATTTCAGTTGATTTAACTATTTTTTCTTTACACCTAGCAGGAAT

TTCATCAATTTTAGGAGCAATTAATTTTATTTGTACAATTCTTAATATAATACCTAATAACATAAAATTAAATCAAATTC

CACTTTTCCCTTGATCAATTTTAATTACAGCTATCTTATTAATTTTATCTTTACCAGTTTTAGCTGGTGCTATTACAATA

TTATTAACTGATCGAAACTTAAATACATCATTTTTTGATCCAGCAGGAGGAGGAGATCCTATTTTATACCAACATTTATT

TTGATTCTTTGGACACCGGG

**GGS-10:**

AACTTTATTTGGTaTTTGATCAGGTATAATTGGATCTTCACTTAGAATTCTAATTCGTTTAGAATTAAGTCAAATTAATT

CTATTATTAACAATAATCAATTATATAATGTAATTGTTACAATTCATGCTTTTATTATAATTTTTTTTATAACTATACCA

ATTGTAATTGGTGGATTTGGAAATTGATTAATTCCTATAATAATAGGATGTCCTGATATATCATTTCCTCGCTTAAATAA

TATTAGATTTTGATTATTACCTCCTTCATTAATAATAATAATTTGCAGTTTCTTAATTAATAATGGAACAGGAACAGGAT

GAACTATTTATCCACCTTTATCAAATAATATTGCACATAATAACATTTCAGTTGATTTAACTATTTTTTCTTTACACCTA

GCAGGAATTTCATCAATTTTAGGAGCAATTAATTTTATTTGTACAATTCTTAATATAATACCTAATAACATAAAATTAAA

TCAAATTCCACTTTTCCCTTGATCAATTTTAATTACAGCTATCTTATTAATTTTATCTTTACCAGTTTTAGCTGGTGCTA

TTACAATATTATTAACTGATCGAAACTTAAATACATCATTTTTTGATCCAGCAGGAGGAGGAGATCCTATTTTATACCAA

CATTTATTTTGATTCTTTGGAACC

**GYN-1:**

CTCTTTGGTaTTTGATCAGGTATAATTGGATCTTCACTTAGAATTCTAATTCGTTTAGAATTAAGTCAAATTAATTCTAT

TATTAACAATAATCAATTATATAATGTAATTGTTACAATTCATGCTTTTATTATAATTTTTTTTATAACTATACCAATTG

TAATTGGTGGATTTGGAAATTGATTAATTCCTATAATAATAGGATGTCCTGATATATCATTTCCTCGCTTAAATAATATT

AGATTTTGATTATTACCTCCTTCATTAATAATAATAATTTGCAGTTTCTTAATTAATAATGGAACAGGAACAGGATGAAC

TATTTATCCACCTTTATCAAATAATATTGCACATAATAACATTTCAGTTGATTTAACTATTTTTTCTTTACACCTAGCAG

GAATTTCATCAATTTTAGGAGCAATTAATTTTATTTGTACAATTCTTAATATAATACCTAATAACATAAAATTAAATCAA

ATTCCACTTTTCCCTTGATCAATTTTAATTACAGCTATCTTATTAATTTTATCTTTACCAGTTTTAGCTGGTGCTATTAC

AATATTATTAACTGATCGAAACTTAAATACATCATTTTTTGATCCAGCAGGAGGAGGAGATCCTATTTTATACCAACATT

TATTTTGATTCTTTGGAAC

**GYN-2:**

CTCATTGGTaTTTGATCAGGTATAATTGGATCTTCACTTAGAATTCTAATTCGTTTAGAATTAAGTCAAATTAATTCTAT

TATTAACAATAATCAATTATATAATGTAATTGTTACAATTCATGCTTTTATTATAATTTTTTTTATAACTATACCAATTG

TAATTGGTGGATTTGGAAATTGATTAATTCCTATAATAATAGGATGTCCTGATATATCATTTCCTCGCTTAAATAATATT

AGATTTTGATTATTACCTCCTTCATTAATAATAATAATTTGCAGTTTCTTAATTAATAATGGAACAGGAACAGGATGAAC

TATTTATCCACCTTTATCAAATAATATTGCACATAATAACATTTCAGTTGATTTAACTATTTTTTCTTTACACCTAGCAG

GAATTTCATCAATTTTAGGAGCAATTAATTTTATTTGTACAATTCTTAATATAATACCTAATAACATAAAATTAAATCAA

ATTCCACTTTTCCCTTGATCAATTTTAATTACAGCTATCTTATTAATTTTATCTTTACCAGTTTTAGCTGGTGCTATTAC

AATATTATTAACTGATCGAAACTTAAATACATCATTTTTTGATCCAGCAGGAGGAGGAGATCCTATTTTATACCAACATT

TATTTTGATTCTTTGGAAC

**GYN-3:**

CCCCTTTGGTatTTGATCAGGTATAATTGGATCTTCACTTAGAATTCTAATTCGTTTAGAATTAAGTCAAATTAATTCTA

TTATTAACAATAATCAATTATATAATGTAATTGTTACAATTCATGCTTTTATTATAATTTTTTTTATAACTATACCAATT

GTAATTGGTGGATTTGGAAATTGATTAATTCCTATAATAATAGGATGTCCTGATATATCATTTCCTCGCTTAAATAATAT

TAGATTTTGATTATTACCTCCTTCATTAATAATAATAATTTGCAGTTTCTTAATTAATAATGGAACAGGAACAGGATGAA

CTATTTATCCACCTTTATCAAATAATATTGCACATAATAACATTTCAGTTGATTTAACTATTTTTTCTTTACACCTAGCA

GGAATTTCATCAATTTTAGGAGCAATTAATTTTATTTGTACAATTCTTAATATAATACCTAATAACATAAAATTAAATCA

AATTCCACTTTTCCCTTGATCAATTTTAATTACAGCTATCTTATTAATTTTATCTTTACCAGTTTTAGCTGGTGCTATTA

CAATATTATTAACTGATCGAAACTTAAATACATCATTTTTTGATCCAGCAGGAGGAGGAGATCCTATTTTATACCAACAT

TTATTTTGATTCTTTGGCAC

**GYN-4:**

CCCCTCGGTaTTTGATCAGGTATAATTGGATCTTCACTTAGAATTCTAATTCGTTTAGAATTAAGTCAAATTAATTCTAT

TATTAACAATAATCAATTATATAATGTAATTGTTACAATTCATGCTTTTATTATAATTTTTTTTATAACTATACCAATTG

TAATTGGTGGATTTGGAAATTGATTAATTCCTATAATAATAGGATGTCCTGATATATCATTTCCTCGCTTAAATAATATT

AGATTTTGATTATTACCTCCTTCATTAATAATAATAATTTGCAGTTTCTTAATTAATAATGGAACAGGAACAGGATGAAC

TATTTATCCACCTTTATCAAATAATATTGCACATAATAACATTTCAGTTGATTTAACTATTTTTTCTTTACACCTAGCAG

GAATTTCATCAATTTTAGGAGCAATTAATTTTATTTGTACAATTCTTAATATAATACCTAATAACATAAAATTAAATCAA

ATTCCACTTTTCCCTTGATCAATTTTAATTACAGCTATCTTATTAATTTTATCTTTACCAGTTTTAGCTGGTGCTATTAC

AATATTATTAACTGATCGAAACTTAAATACATCATTTTTTGATCCAGCAGGAGGAGGAGATCCTATTTTATACCAACATT

TATTTTGATTCTTTGGAACC

**GYN-5:**

TATTGGTaTTTGATCAGGTATAATTGGATCTTCACTTAGAATTCTAATTCGTTTAGAATTAAGTCAAATTAATTCTATTA

TTAACAATAATCAATTATATAATGTAATTGTTACAATTCATGCTTTTATTATAATTTTTTTTATAACTATACCAATTGTA

ATTGGTGGATTTGGAAATTGATTAATTCCTATAATAATAGGATGTCCTGATATATCATTTCCTCGCTTAAATAATATTAG

ATTTTGATTATTACCTCCTTCATTAATAATAATAATTTGCAGTTTCTTAATTAATAATGGAACAGGAACAGGATGAACTA

TTTATCCACCTTTATCAAATAATATTGCACATAATAACATTTCAGTTGATTTAACTATTTTTTCTTTACACCTAGCAGGA

ATTTCATCAATTTTAGGAGCAATTAATTTTATTTGTACAATTCTTAATATAATACCTAATAACATAAAATTAAATCAAAT

TCCACTTTTCCCTTGATCAATTTTAATTACAGCTATCTTATTAATTTTATCTTTACCAGTTTTAGCTGGTGCTATTACAA

TATTATTAACTGATCGAAACTTAAATACATCATTTTTTGATCCAGCAGGAGGAGGAGATCCTATTTTATACCAACATTTA

TTTTGATTCTTTGGAACC

**GYN-6:**

TATTGGTaTTTGATCAGGTATAATTGGATCTTCACTTAGAATTCTAATTCGTTTAGAATTAAGTCAAATTAATTCTATTA

TTAACAATAATCAATTATATAATGTAATTGTTACAATTCATGCTTTTATTATAATTTTTTTTATAACTATACCAATTGTA

ATTGGTGGATTTGGAAATTGATTAATTCCTATAATAATAGGATGTCCTGATATATCATTTCCTCGCTTAAATAATATTAG

ATTTTGATTATTACCTCCTTCATTAATAATAATAATTTGCAGTTTCTTAATTAATAATGGAACAGGAACAGGATGAACTA

TTTATCCACCTTTATCAAATAATATTGCACATAATAACATTTCAGTTGATTTAACTATTTTTTCTTTACACCTAGCAGGA

ATTTCATCAATTTTAGGAGCAATTAATTTTATTTGTACAATTCTTAATATAATACCTAATAACATAAAATTAAATCAAAT

TCCACTTTTCCCTTGATCAATTTTAATTACAGCTATCTTATTAATTTTATCTTTACCAGTTTTAGCTGGTGCTATTACAA

TATTATTAACTGATCGAAACTTAAATACATCATTTTTTGATCCAGCAGGAGGAGGAGATCCTATTTTATACCAACATTTA

TTTTGATTCTTTGGACACCGGG

**GYN-7:**

TTATTTGGTaTTTGATCAGGTATAATTGGATCTTCACTTAGAATTCTAATTCGTTTAGAATTAAGTCAAATTAATTCTAT

TATTAACAATAATCAATTATATAATGTAATTGTTACAATTCATGCTTTTATTATAATTTTTTTTATAACTATACCAATTG

TAATTGGTGGATTTGGAAATTGATTAATTCCTATAATAATAGGATGTCCTGATATATCATTTCCTCGCTTAAATAATATT

AGATTTTGATTATTACCTCCTTCATTAATAATAATAATTTGCAGTTTCTTAATTAATAATGGAACAGGAACAGGATGAAC

TATTTATCCACCTTTATCAAATAATATTGCACATAATAACATTTCAGTTGATTTAACTATTTTTTCTTTACACCTAGCAG

GAATTTCATCAATTTTAGGAGCAATTAATTTTATTTGTACAATTCTTAATATAATACCTAATAACATAAAATTAAATCAA

ATTCCACTTTTCCCTTGATCAATTTTAATTACAGCTATCTTATTAATTTTATCTTTACCAGTTTTAGCTGGTGCTATTAC

AATATTATTAACTGATCGAAACTTAAATACATCATTTTTTGATCCAGCAGGAGGAGGAGATCCTATTTTATACCAACATT

TATTTTGATTCTTTGGCAC

**GYN-8:**

TATTGGTaTTTGATCAGGTATAATTGGATCTTCACTTAGAATTCTAATTCGTTTAGAATTAAGTCAAATTAATTCTATTA

TTAACAATAATCAATTATATAATGTAATTGTTACAATTCATGCTTTTATTATAATTTTTTTTATAACTATACCAATTGTA

ATTGGTGGATTTGGAAATTGATTAATTCCTATAATAATAGGATGTCCTGATATATCATTTCCTCGCTTAAATAATATTAG

ATTTTGATTATTACCTCCTTCATTAATAATAATAATTTGCAGTTTCTTAATTAATAATGGAACAGGAACAGGATGAACTA

TTTATCCACCTTTATCAAATAATATTGCACATAATAACATTTCAGTTGATTTAACTATTTTTTCTTTACACCTAGCAGGA

ATTTCATCAATTTTAGGAGCAATTAATTTTATTTGTACAATTCTTAATATAATACCTAATAACATAAAATTAAATCAAAT

TCCACTTTTCCCTTGATCAATTTTAATTACAGCTATCTTATTAATTTTATCTTTACCAGTTTTAGCTGGTGCTATTACAA

TATTATTAACTGATCGAAACTTAAATACATCATTTTTTGATCCAGCAGGAGGAGGAGATCCTATTTTATACCAACATTTA

TTTTGATTCTTTGGACACCGGG

**GYN-9:**

TTTTGGTaTTTGATCAGGTATAATTGGATCTTCACTTAGAATTCTAATTCGTTTAGAATTAAGTCAAATTAATTCTATTA

TTAACAATAATCAATTATATAATGTAATTGTTACAATTCATGCTTTTATTATAATTTTTTTTATAACTATACCAATTGTA

ATTGGTGGATTTGGAAATTGATTAATTCCTATAATAATAGGATGTCCTGATATATCATTTCCTCGCTTAAATAATATTAG

ATTTTGATTATTACCTCCTTCATTAATAATAATAATTTGCAGTTTCTTAATTAATAATGGAACAGGAACAGGATGAACTA

TTTATCCACCTTTATCAAATAATATTGCACATAATAACATTTCAGTTGATTTAACTATTTTTTCTTTACACCTAGCAGGA

ATTTCATCAATTTTAGGAGCAATTAATTTTATTTGTACAATTCTTAATATAATACCTAATAACATAAAATTAAATCAAAT

TCCACTTTTCCCTTGATCAATTTTAATTACAGCTATCTTATTAATTTTATCTTTACCAGTTTTAGCTGGTGCTATTACAA

TATTATTAACTGATCGAAACTTAAATACATCATTTTTTGATCCAGCAGGAGGAGGAGATCCTATTTTATACCAACATTTA

TTTTGATTCTTTGGACACCGG

**RGS-1:**

CTGGTaTTTGATCAGGTATAATTGGATCTTCACTTAGAATTCTAATTCGTTTAGAATTAAGTCAAATTAATTCTATTATT

AACAATAATCAATTATATAATGTAATTGTTACAATTCATGCTTTTATTATAATTTTTTTTATAACTATACCAATTGTAAT

TGGTGGATTTGGAAATTGATTAATTCCTATAATAATAGGATGTCCTGATATATCATTTCCTCGCTTAAATAATATTAGAT

TTTGATTATTACCTCCTTCATTAATAATAATAATTTGCAGTTTCTTAATTAATAATGGAACAGGAACAGGATGAACTATT

TATCCACCTTTATCAAATAATATTGCACATAATAACATTTCAGTTGATTTAACTATTTTTTCTTTACACCTAGCAGGAAT

TTCATCAATTTTAGGAGCAATTAATTTTATTTGTACAATTCTTAATATAATACCTAATAACATAAAATTAAATCAAATTC

CACTTTTCCCTTGATCAATTTTAATTACAGCTATCTTATTAATTTTATCTTTACCAGTTTTAGCTGGTGCTATTACAATA

TTATTAACTGATCGAAACTTAAATACATCATTTTTTGATCCAGCAGGAGGAGGAGATCCTATTTTATACCAACATTTATT

TTGATTCTTTGGAACC

**RGS-2:**

AACTTTATTGGTaTTTGATCAGGTATAATTGGATCTTCACTTAGAATTCTAATTCGTTTAGAATTAAGTCAAATTAATTC

TATTATTAACAATAATCAATTATATAATGTAATTGTTACAATTCATGCTTTTATTATAATTTTTTTTATAACTATACCAA

TTGTAATTGGTGGATTTGGAAATTGATTAATTCCTATAATAATAGGATGTCCTGATATATCATTTCCTCGCTTAAATAAT

ATTAGATTTTGATTATTACCTCCTTCATTAATAATAATAATTTGCAGTTTCTTAATTAATAATGGAACAGGAACAGGATG

AACTATTTATCCACCTTTATCAAATAATATTGCACATAATAACATTTCAGTTGATTTAACTATTTTTTCTTTACACCTAG

CAGGAATTTCATCAATTTTAGGAGCAATTAATTTTATTTGTACAATTCTTAATATAATACCTAATAACATAAAATTAAAT

CAAATTCCACTTTTCCCTTGATCAATTTTAATTACAGCTATCTTATTAATTTTATCTTTACCAGTTTTAGCTGGTGCTAT

TACAATATTATTAACTGATCGAAACTTAAATACATCATTTTTTGATCCAGCAGGAGGAGGAGATCCTATTTTATACCAAC

ATTTATTTTGATTCTTTGGAACC

**RGS-3:**

ATGGTaTTTGATCAGGTATAATTGGATCTTCACTTAGAATTCTAATTCGTTTAGAATTAAGTCAAATTAATTCTATTATT

AACAATAATCAATTATATAATGTAATTGTTACAATTCATGCTTTTATTATAATTTTTTTTATAACTATACCAATTGTAAT

TGGTGGATTTGGAAATTGATTAATTCCTATAATAATAGGATGTCCTGATATATCATTTCCTCGCTTAAATAATATTAGAT

TTTGATTATTACCTCCTTCATTAATAATAATAATTTGCAGTTTCTTAATTAATAATGGAACAGGAACAGGATGAACTATT

TATCCACCTTTATCAAATAATATTGCACATAATAACATTTCAGTTGATTTAACTATTTTTTCTTTACACCTAGCAGGAAT

TTCATCAATTTTAGGAGCAATTAATTTTATTTGTACAATTCTTAATATAATACCTAATAACATAAAATTAAATCAAATTC

CACTTTTCCCTTGATCAATTTTAATTACAGCTATCTTATTAATTTTATCTTTACCAGTTTTAGCTGGTGCTATTACAATA

TTATTAACTGATCGAAACTTAAATACATCATTTTTTGATCCAGCAGGAGGAGGAGATCCTATTTTATACCAACATTTATT

TTGATTCTTTGGACA

**RGS-4:**

ATTGGTaTTTGATCAGGTATAATTGGATCTTCACTTAGAATTCTAATTCGTTTAGAATTAAGTCAAATTAATTCTATTAT

TAACAATAATCAATTATATAATGTAATTGTTACAATTCATGCTTTTATTATAATTTTTTTTATAACTATACCAATTGTAA

TTGGTGGATTTGGAAATTGATTAATTCCTATAATAATAGGATGTCCTGATATATCATTTCCTCGCTTAAATAATATTAGA

TTTTGATTATTACCTCCTTCATTAATAATAATAATTTGCAGTTTCTTAATTAATAATGGAACAGGAACAGGATGAACTAT

TTATCCACCTTTATCAAATAATATTGCACATAATAACATTTCAGTTGATTTAACTATTTTTTCTTTACACCTAGCAGGAA

TTTCATCAATTTTAGGAGCAATTAATTTTATTTGTACAATTCTTAATATAATACCTAATAACATAAAATTAAATCAAATT

CCACTTTTCCCTTGATCAATTTTAATTACAGCTATCTTATTAATTTTATCTTTACCAGTTTTAGCTGGTGCTATTACAAT

ATTATTAACTGATCGAAACTTAAATACATCATTTTTTGATCCAGCAGGAGGAGGAGATCCTATTTTATACCAACATTTAT

TTTGATTCTTTGGACAC

**RGS-5:**

CTTTATTTGGTaTTTGATCAGGTATAATTGGATCTTCACTTAGAATTCTAATTCGTTTAGAATTAAGTCAAATTAATTCT

ATTATTAACAATAATCAATTATATAATGTAATTGTTACAATTCATGCTTTTATTATAATTTTTTTTATAACTATACCAAT

TGTAATTGGTGGATTTGGAAATTGATTAATTCCTATAATAATAGGATGTCCTGATATATCATTTCCTCGCTTAAATAATA

TTAGATTTTGATTATTACCTCCTTCATTAATAATAATAATTTGCAGTTTCTTAATTAATAATGGAACAGGAACAGGATGA

ACTATTTATCCACCTTTATCAAATAATATTGCACATAATAACATTTCAGTTGATTTAACTATTTTTTCTTTACACCTAGC

AGGAATTTCATCAATTTTAGGAGCAATTAATTTTATTTGTACAATTCTTAATATAATACCTAATAACATAAAATTAAATC

AAATTCCACTTTTCCCTTGATCAATTTTAATTACAGCTATCTTATTAATTTTATCTTTACCAGTTTTAGCTGGTGCTATT

ACAATATTATTAACTGATCGAAACTTAAATACATCATTTTTTGATCCAGCAGGAGGAGGAGATCCTATTTTATACCAACA

TTTATTTTGATTCTTTGGACATCCTGGGAAGTTAAA

**RGS-6:**

CTCTTTATTGGTaTTTGATCAGGTATAATTGGATCTTCACTTAGAATTCTAATTCGTTTAGAATTAAGTCAAATTAATTC

TATTATTAACAATAATCAATTATATAATGTAATTGTTACAATTCATGCTTTTATTATAATTTTTTTTATAACTATACCAA

TTGTAATTGGTGGATTTGGAAATTGATTAATTCCTATAATAATAGGATGTCCTGATATATCATTTCCTCGCTTAAATAAT

ATTAGATTTTGATTATTACCTCCTTCATTAATAATAATAATTTGCAGTTTCTTAATTAATAATGGAACAGGAACAGGATG

AACTATTTATCCACCTTTATCAAATAATATTGCACATAATAACATTTCAGTTGATTTAACTATTTTTTCTTTACACCTAG

CAGGAATTTCATCAATTTTAGGAGCAATTAATTTTATTTGTACAATTCTTAATATAATACCTAATAACATAAAATTAAAT

CAAATTCCACTTTTCCCTTGATCAATTTTAATTACAGCTATCTTATTAATTTTATCTTTACCAGTTTTAGCTGGTGCTAT

TACAATATTATTAACTGATCGAAACTTAAATACATCATTTTTTGATCCAGCAGGAGGAGGAGATCCTATTTTATACCAAC

ATTTATTTTGATTCTTTGGCAC

**RGS-7:**

CCAACTTTATTGGTaTTTGATCAGGTATAATTGGATCTTCACTTAGAATTCTAATTCGTTTAGAATTAAGTCAAATTAAT

TCTATTATTAACAATAATCAATTATATAATGTAATTGTTACAATTCATGCTTTTATTATAATTTTTTTTATAACTATACC

AATTGTAATTGGTGGATTTGGAAATTGATTAATTCCTATAATAATAGGATGTCCTGATATATCATTTCCTCGCTTAAATA

ATATTAGATTTTGATTATTACCTCCTTCATTAATAATAATAATTTGCAGTTTCTTAATTAATAATGGAACAGGAACAGGA

TGAACTATTTATCCACCTTTATCAAATAATATTGCACATAATAACATTTCAGTTGATTTAACTATTTTTTCTTTACACCT

AGCAGGAATTTCATCAATTTTAGGAGCAATTAATTTTATTTGTACAATTCTTAATATAATACCTAATAACATAAAATTAA

ATCAAATTCCACTTTTCCCTTGATCAATTTTAATTACAGCTATCTTATTAATTTTATCTTTACCAGTTTTAGCTGGTGCT

ATTACAATATTATTAACTGATCGAAACTTAAATACATCATTTTTTGATCCAGCAGGAGGAGGAGATCCTATTTTATACCA

ACATTTATTTTGATTCTTTGGACATCTT

**RGS-8:**

CTTTATTTGGTaTTTGATCAGGTATAATTGGATCTTCACTTAGAATTCTAATTCGTTTAGAATTAAGTCAAATTAATTCT

ATTATTAACAATAATCAATTATATAATGTAATTGTTACAATTCATGCTTTTATTATAATTTTTTTTATAACTATACCAAT

TGTAATTGGTGGATTTGGAAATTGATTAATTCCTATAATAATAGGATGTCCTGATATATCATTTCCTCGCTTAAATAATA

TTAGATTTTGATTATTACCTCCTTCATTAATAATAATAATTTGCAGTTTCTTAATTAATAATGGAACAGGAACAGGATGA

ACTATTTATCCACCTTTATCAAATAATATTGCACATAATAACATTTCAGTTGATTTAACTATTTTTTCTTTACACCTAGC

AGGAATTTCATCAATTTTAGGAGCAATTAATTTTATTTGTACAATTCTTAATATAATACCTAATAACATAAAATTAAATC

AAATTCCACTTTTCCCTTGATCAATTTTAATTACAGCTATCTTATTAATTTTATCTTTACCAGTTTTAGCTGGTGCTATT

ACAATATTATTAACTGATCGAAACTTAAATACATCATTTTTTGATCCAGCAGGAGGAGGAGATCCTATTTTATACCAACA

TTTATTTTGATTCTTTGGACATCTTTGAAAGGTTTAA

**RGS-9:**

CTTGGTaTTTGATCAGGTATAATTGGATCTTCACTTAGAATTCTAATTCGTTTAGAATTAAGTCAAATTAATTCTATTAT

TAACAATAATCAATTATATAATGTAATTGTTACAATTCATGCTTTTATTATAATTTTTTTTATAACTATACCAATTGTAA

TTGGTGGATTTGGAAATTGATTAATTCCTATAATAATAGGATGTCCTGATATATCATTTCCTCGCTTAAATAATATTAGA

TTTTGATTATTACCTCCTTCATTAATAATAATAATTTGCAGTTTCTTAATTAATAATGGAACAGGAACAGGATGAACTAT

TTATCCACCTTTATCAAATAATATTGCACATAATAACATTTCAGTTGATTTAACTATTTTTTCTTTACACCTAGCAGGAA

TTTCATCAATTTTAGGAGCAATTAATTTTATTTGTACAATTCTTAATATAATACCTAATAACATAAAATTAAATCAAATT

CCACTTTTCCCTTGATCAATTTTAATTACAGCTATCTTATTAATTTTATCTTTACCAGTTTTAGCTGGTGCTATTACAAT

ATTATTAACTGATCGAAACTTAAATACATCATTTTTTGATCCAGCAGGAGGAGGAGATCCTATTTTATACCAACATTTAT

TTTGATTCTTTGGACACCGG

**RGS-10:**

TATTGGTaTTTGATCAGGTATAATTGGATCTTCACTTAGAATTCTAATTCGTTTAGAATTAAGTCAAATTAATTCTATTA

TTAACAATAATCAATTATATAATGTAATTGTTACAATTCATGCTTTTATTATAATTTTTTTTATAACTATACCAATTGTA

ATTGGTGGATTTGGAAATTGATTAATTCCTATAATAATAGGATGTCCTGATATATCATTTCCTCGCTTAAATAATATTAG

ATTTTGATTATTACCTCCTTCATTAATAATAATAATTTGCAGTTTCTTAATTAATAATGGAACAGGAACAGGATGAACTA

TTTATCCACCTTTATCAAATAATATTGCACATAATAACATTTCAGTTGATTTAACTATTTTTTCTTTACACCTAGCAGGA

ATTTCATCAATTTTAGGAGCAATTAATTTTATTTGTACAATTCTTAATATAATACCTAATAACATAAAATTAAATCAAAT

TCCACTTTTCCCTTGATCAATTTTAATTACAGCTATCTTATTAATTTTATCTTTACCAGTTTTAGCTGGTGCTATTACAA

TATTATTAACTGATCGAAACTTAAATACATCATTTTTTGATCCAGCAGGAGGAGGAGATCCTATTTTATACCAACATTTA

TTTTGATTCTTTGGCA

**RQH-2:**

AACTTTATTTGGTaaTTTGATCAGGTATAATTGGATCTTCACTTAGAATTCTAATTCGTTTAGAATTAAGTCAAATTAAT

TCTATTATTAACAATAATCAATTATATAATGTAATTGTTACAATTCATGCTTTTATTATAATTTTTTTTATAACTATACC

AATTGTAATTGGTGGATTTGGAAATTGATTAATTCCTATAATAATAGGATGTCCTGATATATCATTTCCTCGCTTAAATA

ATATTAGATTTTGATTATTACCTCCTTCATTAATAATAATAATTTGCAGTTTCTTAATTAATAATGGAACAGGAACAGGA

TGAACTATTTATCCACCTTTATCAAATAATATTGCACATAATAACATTTCAGTTGATTTAACTATTTTTTCTTTACACCT

AGCAGGAATTTCATCAATTTTAGGAGCAATTAATTTTATTTGTACAATTCTTAATATAATACCTAATAACATAAAATTAA

ATCAAATTCCACTTTTCCCTTGATCAATTTTAATTACAGCTATCTTATTAATTTTATCTTTACCAGTTTTAGCTGGTGCT

ATTACAATATTATTAACTGATCGAAACTTAAATACATCATTTTTTGATCCAGCAGGAGGAGGAGATCCTATTTTATACCA

ACATTTATTTTGATTCTTTGGACATCCTGGA

**RQH-3:**

CTTTATTTGGTaTTTGATCAGGTATAATTGGATCTTCACTTAGAATTCTAATTCGTTTAGAATTAAGTCAAATTAATTCT

ATTATTAACAATAATCAATTATATAATGTAATTGTTACAATTCATGCTTTTATTATAATTTTTTTTATAACTATACCAAT

TGTAATTGGTGGATTTGGAAATTGATTAATTCCTATAATAATAGGATGTCCTGATATATCATTTCCTCGCTTAAATAATA

TTAGATTTTGATTATTACCTCCTTCATTAATAATAATAATTTGCAGTTTCTTAATTAATAATGGAACAGGAACAGGATGA

ACTATTTATCCACCTTTATCAAATAATATTGCACATAATAACATTTCAGTTGATTTAACTATTTTTTCTTTACACCTAGC

AGGAATTTCATCAATTTTAGGAGCAATTAATTTTATTTGTACAATTCTTAATATAATACCTAATAACATAAAATTAAATC

AAATTCCACTTTTCCCTTGATCAATTTTAATTACAGCTATCTTATTAATTTTATCTTTACCAGTTTTAGCTGGTGCTATT

ACAATATTATTAACTGATCGAAACTTAAATACATCATTTTTTGATCCAGCAGGAGGAGGAGATCCTATTTTATACCAACA

TTTATTTTGATTCTTTGGAAC

**RQH-4:**

TTTTATTGGTaTTTGATCAGGTATAATTGGATCTTCACTTAGAATTCTAATTCGTTTAGAATTAAGTCAAATTAATTCTA

TTATTAACAATAATCAATTATATAATGTAATTGTTACAATTCATGCTTTTATTATAATTTTTTTTATAACTATACCAATT

GTAATTGGTGGATTTGGAAATTGATTAATTCCTATAATAATAGGATGTCCTGATATATCATTTCCTCGCTTAAATAATAT

TAGATTTTGATTATTACCTCCTTCATTAATAATAATAATTTGCAGTTTCTTAATTAATAATGGAACAGGAACAGGATGAA

CTATTTATCCACCTTTATCAAATAATATTGCACATAATAACATTTCAGTTGATTTAACTATTTTTTCTTTACACCTAGCA

GGAATTTCATCAATTTTAGGAGCAATTAATTTTATTTGTACAATTCTTAATATAATACCTAATAACATAAAATTAAATCA

AATTCCACTTTTCCCTTGATCAATTTTAATTACAGCTATCTTATTAATTTTATCTTTACCAGTTTTAGCTGGTGCTATTA

CAATATTATTAACTGATCGAAACTTAAATACATCATTTTTTGATCCAGCAGGAGGAGGAGATCCTATTTTATACCAACAT

TTATTTTGATTCTTTGGCA

**RQH-5:**

CATTGGTaTTTGATCAGGTATAATTGGATCTTCACTTAGAATTCTAATTCGTTTAGAATTAAGTCAAATTAATTCTATTA

TTAACAATAATCAATTATATAATGTAATTGTTACAATTCATGCTTTTATTATAATTTTTTTTATAACTATACCAATTGTA

ATTGGTGGATTTGGAAATTGATTAATTCCTATAATAATAGGATGTCCTGATATATCATTTCCTCGCTTAAATAATATTAG

ATTTTGATTATTACCTCCTTCATTAATAATAATAATTTGCAGTTTCTTAATTAATAATGGAACAGGAACAGGATGAACTA

TTTATCCACCTTTATCAAATAATATTGCACATAATAACATTTCAGTTGATTTAACTATTTTTTCTTTACACCTAGCAGGA

ATTTCATCAATTTTAGGAGCAATTAATTTTATTTGTACAATTCTTAATATAATACCTAATAACATAAAATTAAATCAAAT

TCCACTTTTCCCTTGATCAATTTTAATTACAGCTATCTTATTAATTTTATCTTTACCAGTTTTAGCTGGTGCTATTACAA

TATTATTAACTGATCGAAACTTAAATACATCATTTTTTGATCCAGCAGGAGGAGGAGATCCTATTTTATACCAACATTTA

TTTTGATTCTTTGGAACC

**RQH-6:**

TTTGGTaTTTGATCAGGTATAATTGGATCTTCACTTAGAATTCTAATTCGTTTAGAATTAAGTCAAATTAATTCTATTAT

TAACAATAATCAATTATATAATGTAATTGTTACAATTCATGCTTTTATTATAATTTTTTTTATAACTATACCAATTGTAA

TTGGTGGATTTGGAAATTGATTAATTCCTATAATAATAGGATGTCCTGATATATCATTTCCTCGCTTAAATAATATTAGA

TTTTGATTATTACCTCCTTCATTAATAATAATAATTTGCAGTTTCTTAATTAATAATGGAACAGGAACAGGATGAACTAT

TTATCCACCTTTATCAAATAATATTGCACATAATAACATTTCAGTTGATTTAACTATTTTTTCTTTACACCTAGCAGGAA

TTTCATCAATTTTAGGAGCAATTAATTTTATTTGTACAATTCTTAATATAATACCTAATAACATAAAATTAAATCAAATT

CCACTTTTCCCTTGATCAATTTTAATTACAGCTATCTTATTAATTTTATCTTTACCAGTTTTAGCTGGTGCTATTACAAT

ATTATTAACTGATCGAAACTTAAATACATCATTTTTTGATCCAGCAGGAGGAGGAGATCCTATTTTATACCAACATTTAT

TTTGATTCTTTGGCACC

**RQH-7:**

TTTGGTaTTTGATCAGGTATAATTGGATCTTCACTTAGAATTCTAATTCGTTTAGAATTAAGTCAAATTAATTCTATTAT

TAACAATAATCAATTATATAATGTAATTGTTACAATTCATGCTTTTATTATAATTTTTTTTATAACTATACCAATTGTAA

TTGGTGGATTTGGAAATTGATTAATTCCTATAATAATAGGATGTCCTGATATATCATTTCCTCGCTTAAATAATATTAGA

TTTTGATTATTACCTCCTTCATTAATAATAATAATTTGCAGTTTCTTAATTAATAATGGAACAGGAACAGGATGAACTAT

TTATCCACCTTTATCAAATAATATTGCACATAATAACATTTCAGTTGATTTAACTATTTTTTCTTTACACCTAGCAGGAA

TTTCATCAATTTTAGGAGCAATTAATTTTATTTGTACAATTCTTAATATAATACCTAATAACATAAAATTAAATCAAATT

CCACTTTTCCCTTGATCAATTTTAATTACAGCTATCTTATTAATTTTATCTTTACCAGTTTTAGCTGGTGCTATTACAAT

ATTATTAACTGATCGAAACTTAAATACATCATTTTTTGATCCAGCAGGAGGAGGAGATCCTATTTTATACCAACATTTAT

TTTGATTCTTTGGCACC

**RQH-8:**

CTTGGTTTTGATCAGGTATAATTGGATCTTCACTTAGAATTCTAATTCGTTTAGAATTAAGTCAAATTAATTCTATTATT

AACAATAATCAATTATATAATGTAATTGTTACAATTCATGCTTTTATTATAATTTTTTTTATAACTATACCAATTGTAAT

TGGTGGATTTGGAAATTGATTAATTCCTATAATAATAGGATGTCCTGATATATCATTTCCTCGCTTAAATAATATTAGAT

TTTGATTATTACCTCCTTCATTAATAATAATAATTTGCAGTTTCTTAATTAATAATGGAACAGGAACAGGATGAACTATT

TATCCACCTTTATCAAATAATATTGCACATAATAACATTTCAGTTGATTTAACTATTTTTTCTTTACACCTAGCAGGAAT

TTCATCAATTTTAGGAGCAATTAATTTTATTTGTACAATTCTTAATATAATACCTAATAACATAAAATTAAATCAAATTC

CACTTTTCCCTTGATCAATTTTAATTACAGCTATCTTATTAATTTTATCTTTACCAGTTTTAGCTGGTGCTATTACAATA

TTATTAACTGATCGAAACTTAAATACATCATTTTTTGATCCAGCAGGAGGAGGAGATCCTATTTTATACCAACATTTATT

TTGATTCTTTGGCCC

**RQH-9:**

AACTTTATTGGTaTTTGATCAGGTATAATTGGATCTTCACTTAGAATTCTAATTCGTTTAGAATTAAGTCAAATTAATTC

TATTATTAACAATAATCAATTATATAATGTAATTGTTACAATTCATGCTTTTATTATAATTTTTTTTATAACTATACCAA

TTGTAATTGGTGGATTTGGAAATTGATTAATTCCTATAATAATAGGATGTCCTGATATATCATTTCCTCGCTTAAATAAT

ATTAGATTTTGATTATTACCTCCTTCATTAATAATAATAATTTGCAGTTTCTTAATTAATAATGGAACAGGAACAGGATG

AACTATTTATCCACCTTTATCAAATAATATTGCACATAATAACATTTCAGTTGATTTAACTATTTTTTCTTTACACCTAG

CAGGAATTTCATCAATTTTAGGAGCAATTAATTTTATTTGTACAATTCTTAATATAATACCTAATAACATAAAATTAAAT

CAAATTCCACTTTTCCCTTGATCAATTTTAATTACAGCTATCTTATTAATTTTATCTTTACCAGTTTTAGCTGGTGCTAT

TACAATATTATTAACTGATCGAAACTTAAATACATCATTTTTTGATCCAGCAGGAGGAGGAGATCCTATTTTATACCAAC

ATTTATTTTGATTCTTTGGCAC

**RQH-10:**

TTTGGTaTTTGATCAGGTATAATTGGATCTTCACTTAGAATTCTAATTCGTTTAGAATTAAGTCAAATTAATTCTATTAT

TAACAATAATCAATTATATAATGTAATTGTTACAATTCATGCTTTTATTATAATTTTTTTTATAACTATACCAATTGTAA

TTGGTGGATTTGGAAATTGATTAATTCCTATAATAATAGGATGTCCTGATATATCATTTCCTCGCTTAAATAATATTAGA

TTTTGATTATTACCTCCTTCATTAATAATAATAATTTGCAGTTTCTTAATTAATAATGGAACAGGAACAGGATGAACTAT

TTATCCACCTTTATCAAATAATATTGCACATAATAACATTTCAGTTGATTTAACTATTTTTTCTTTACACCTAGCAGGAA

TTTCATCAATTTTAGGAGCAATTAATTTTATTTGTACAATTCTTAATATAATACCTAATAACATAAAATTAAATCAAATT

CCACTTTTCCCTTGATCAATTTTAATTACAGCTATCTTATTAATTTTATCTTTACCAGTTTTAGCTGGTGCTATTACAAT

ATTATTAACTGATCGAAACTTAAATACATCATTTTTTGATCCAGCAGGAGGAGGAGATCCTATTTTATACCAACATTTAT

TTTGATTCTTTGGACACCGTGAAAGTTTAA
